# In Vitro Evaluation of the Safety and Antineoplastic Effects in Gastrointestinal Tumors of Nanostructured Lipid Carriers Loaded with Berberine

**DOI:** 10.3390/pharmaceutics17030331

**Published:** 2025-03-04

**Authors:** Denitsa Stefanova, Yordan Yordanov, Radostina Bogdanova, Christina Voycheva, Borislav Tzankov, Teodora Popova, Magdalena Kondeva-Burdina, Virginia Tzankova, Natalia Toncheva-Moncheva, Diana Tzankova, Marta Slavkova

**Affiliations:** 1 Department of Pharmacology, Pharmacotherapy and Toxicology, Faculty of Pharmacy, Medical University of Sofia, 1000 Sofia, Bulgaria; denitsa.stefanova@pharmfac.mu-sofia.bg (D.S.); yyordanov@pharmfac.mu-sofia.bg (Y.Y.); radostina.bogdanovaa@gmail.com (R.B.); mkondeva@pharmfac.mu-sofia.bg (M.K.-B.); vtzankova@pharmfac.mu-sofia.bg (V.T.); 2 Department of Pharmaceutical Technology and Biopharmaceutics, Faculty of Pharmacy, Medical University of Sofia, 1000 Sofia, Bulgaria; btzankov@pharmfac.mu-sofia.bg (B.T.); tpopova@pharmfac.mu-sofia.bg (T.P.); mslavkova@pharmfac.mu-sofia.bg (M.S.); 3 Institute of Polymers, Bulgarian Academy of Sciences, bl.103 Akad. G. Bonchev Str., 1113 Sofia, Bulgaria; ntoncheva@polymer.bas.bg; 4 Department of Pharmaceutical Chemistry, Faculty of Pharmacy, Medical University—Sofia, 1000 Sofia, Bulgaria; d.tsankova@pharmfac.mu-sofia.bg

**Keywords:** berberine, nanostructured lipid carriers, cholangiocarcinoma, cytotoxicity, genotoxicity

## Abstract

**Background/Objectives**: Natural substances have been a widely studied source of both pharmaceutical excipients and drugs. Berberine (BRB) is a benzylisoquinoline alkaloid isolated from different plant sources. It possesses various pharmacological properties including antibacterial, antitumor, antidiabetic, neuroprotective, hepatoprotective, anti-inflammatory, antioxidant, etc. However, the limited aqueous solubility hinders its application. Nanosized drug delivery systems are an innovative approach for addressing various challenges regarding drug delivery via different routes of administration. Their utilization could improve the solubility of active constituents. **Methods**: A melt-emulsification and ultrasonication technique was applied for the preparation of nanostructured lipid carriers (NLCs). They were thoroughly physicochemically characterized by the means of Dynamic Light Scattering, TEM, FTIR, DSC, TGA, and In Vitro release. The In Vitro efficacy and safety were evaluated on cholangiocarcinoma, colorectal adenocarcinoma, hepatocellular carcinoma, lymphoma, fibroblast, and cardioblast cells, as well as rat liver microsomes by means of cytotoxicity assays and the comet assay. **Results**: The obtained nanoparticles had a spherical shape and size around 158.2 ± 1.8 nm with negative zeta potential. They revealed successful drug loading and improved dissolution of berberine in physiological conditions. The In Vitro safety studies showed that loading BRB in NLCs resulted in improved or retained cytotoxicity to tumor cell lines and reduced cytotoxicity to normal cell lines and liver microsomes. The NLC itself increased microsomal malondialdehyde (MDA) and comet formation. **Conclusions**: A successful preparation of NLCs with berberine is presented. The nanocarriers show favorable physicochemical and biopharmaceutical properties. The cellular experiments show that the NLC loading of berberine could improve its anticancer efficacy and safety. These findings highlight the potential applicability of berberine in gastrointestinal neoplasms and build the foundation for future practical translation.

## 1. Introduction

Every year, almost 4.1 million people are diagnosed with gastrointestinal cancers. The late detection of this type of cancer causes approximately 3 million fatalities annually [1]. As the most common gastrointestinal cancers, colorectal carcinoma and gastric cancer are a significant public health concern due to their high mortality rates. First-line drug therapies often demonstrate limited efficacy. This enforces the inclusion of additional agents, such as biologically active substances of natural origin, alongside primary therapies to enhance treatment effectiveness [2]. Berberine, a benzylisoquinoline natural alkaloid traditionally used in Chinese folk medicine, has garnered significant interest in recent years for its potential in treating malignant tumors [3]. It exhibits a wide range of pharmacological effects, including anti-inflammatory, antioxidant, antihyperglycemic, antihyperlipidemic, neuroprotective, and antiproliferative activities [4]. Some of the mechanisms through which berberine exerts its antineoplastic activity include the induction of programmed cell death (both caspase-dependent and caspase-independent), regulation of miRNA, modulation of various signaling pathways involved in cell proliferation, metastasis, and angiogenesis, as well as its anti-inflammatory and antioxidant properties [5]. However, the main limitation for its practical application is the low bioavailability (under 1%), which is a result of its low solubility and permeability [6]. This ranks it as a class IV drug according to the Biopharmaceutical Classification System (BCS) [7]. It is also a substrate for the P-glycoprotein efflux system [3]. The very low bioavailability and rapid elimination of berberine is associated with the need for high doses in order to exert any therapeutic effect. However, this is associated with undesired immunotoxicity, neurotoxicity, cardiotoxicity, and others [8,9]. Simultaneously some formulation hurdles also arise due to pH- and temperature-dependent solubility and stability [10]. Numerous studies were recently conducted to resolve some of the issues mentioned [11,12,13]. Nanosized drug delivery systems are an innovative approach for elucidating a number of predicaments regarding the drug delivery by different routes of administration. Their utilization could improve the solubility of active pharmaceutical ingredients [14], could increase their bioavailability [15] and stability [16] and can provide targeted or/and stimulus-triggered drug delivery [17]. Solid tumors can be targeted through mechanisms as enhanced permeability and retention or active transport and retention [18,19]. Improvement of the oral bioavailability of berberine was investigated with various formulations, including nanoscale dosage forms, nanocrystalline forms, liposomes, micelles, and microemulsions [20]. Not only do they allow tumor targeting, but can also improve safety by affecting the specific pharmacokinetic profiles of the loaded drugs, as evidenced by the marketed liposomal doxorubicin preparations [21]. Lipid-based nanoparticulate carriers provide favorable biocompatibility and biodegradability [17] while solid lipid nanoparticles (SLNs) and nanostructured lipid carriers (NLC) also offer scalability [22]. NLCs are second generation lipid nanoparticles composed of a blend of liquid and solid lipids at room and body temperatures, along with a surfactant. NLCs were specifically chosen in our study over other nanocarriers due to their excellent biodegradability and biocompatibility as they are GRAS (generally regarded as safe) materials together with high drug loading capability [17,23,24]. Their industrial production is considered more feasible than others [17,25] while it is possible to avoid the use of organic solvents [26]. The constituents needed for their preparation are more affordable than ionizable lipids or some phospholipids used in the preparation of liposomes or lipid nanoparticles (LNPs) [27,28]. Furthermore, NLCs are preferable carriers for drugs belonging to class II and class IV of the BCS as they can affect both their solubility and permeability [29]. There are several proposed mechanisms by which lipid nanocarriers enhance oral permeability. On the one hand, it is related to the improved drug stability in gastrointestinal fluids; on the other, the presence of lipids stimulates bile acids and cholesterol secretion, which is responsible for improved solubilization and uptake. It is supposed that lymphatic transport also plays significant role [30]. In comparison to polymeric nanoparticles, NLCs are considered safer as the lipids they are composed of can be metabolized by typical biological systems [17,30]. Even though lipids have GRAS status, this does not explicitly mean that the nanocarriers would be safe as well. The regulatory bodies (FDA and EMA) recommend extensive In Vitro studies to prove the applicability, efficacy and safety of the nanoparticulate drug delivery systems via different routes of administration [31]. The presence of surfactants is one of the possible safety concern that needs to be addressed [30]. Furthermore, there are some data about the NLC capability of generation of reactive oxygen species (ROS), which are further associated with inflammatory responses [32]. However, there is still limited evidence on their general safety. Hence, it is paramount to carry out research on their inherent potential side effects or as a drug carrier. On the other hand, the existing literature suggests that the pro-oxidant effects of berberine play a significant role in inducing endoplasmic reticulum stress, oxidative stress, and lipid peroxidation, which may contribute to cellular dysfunction and toxicity. A deeper understanding of these interactions could provide valuable insights into berberine’s potential therapeutic applications and safety profile, particularly when formulated in NLC [33].

The aim of the current work was to investigate the preparation and physicochemical, In Vitro pharmacological and toxicological characterization of NLC with berberine as a strategy to implement possible application of the natural compound berberine in the amelioration of gastro-intestinal diseases, especially cancers.

## 2. Materials and Methods

### 2.1. Materials

Berberine hydrochloride (BRB) was purchased from Fluorochem (Hadfield, UK); Caprylic/capric triglyceride (Mygliol^®^ 812N) were kind gift of IOI Oleo GmbH, Hamburg, Germany. Glyceryl palmitostearate (Precirol^®^ 5 ATO) and glyceryl behenate (Compritol^®^ ATO 888) were gifted from Gattefosse (Saint-Priest, France). Oleic acid, castor oil, Tween 20, Tween 80 and Tween 85 were purchased from Sigma Aldrich (Merck KGaA, Darmstadt, Germany). All other excipients used were of analytical grade and were used as received. Purified water was prepared in the laboratory by distillation.

### 2.2. Methods

#### 2.2.1. Nanoparticle Preparation

Prior to NLC preparation, the optimal composition was determined based on the solubility of BRB in several lipids and surfactants. In order to establish the solubility in the liquid lipids (Mygliol^®^ 812N, oleic acid and castor oil) and the surfactants (Tween 20, Tween 80 and Tween 85), an excess of the drug (~0.5 g) was added to 0.5 mL of the lipid and incubated over 24 h in thermostatic shaking water bath at 37 ± 0.5 °C [34]. The samples were centrifuged at 15,000 rpm for 15 min (15,100× *g*) at room temperature (D2012, DragonLab, Beijing, China). A predetermined amount of the supernatant (50 µL) was diluted with methanol (1 mL) and the amount of BRB was spectrophotometrically determined at 430 nm wavelength with the help of spectrophotometer (Thermo Scientific Evolution 300, Madison, WI, USA) based on pre-determined calibration curve.

In the case of solid lipids (Precirol^®^ 5 ATO, Compritol^®^ ATO 888), the lipid (1 g) was melted at 5–10 °C above their melting point. An increment amount of BRB (1–2 mg) was stepwise added to the molten solid lipid and magnetically stirred each time for 1 h until no more undissolved particles could be visually detected [35].

**Figure 1:**
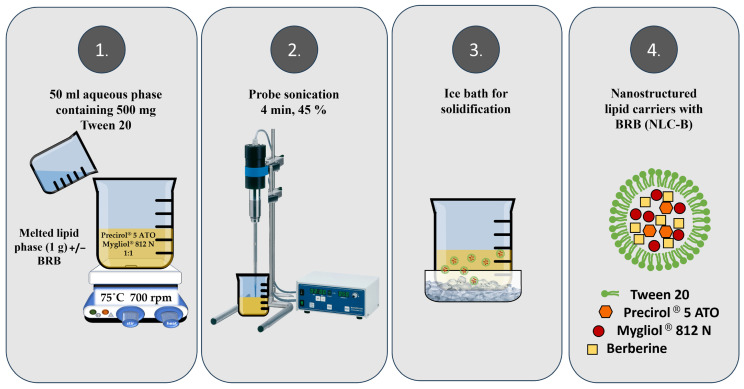
Schematic illustration of the nanoparticle preparation.

Then, the optimal excipients were used for the nanoparticles’ batch preparation following the melt-emulsification and ultrasonication technique as schematically presented on Figure 1. The method was based on the procedure proposed by Sakellari et al. [35] and by Raju et al. [34] with some alterations. Briefly, the lipid phase composed of 500 mg Precirol^®^ 5 ATO and 500 mg Mygliol^®^ 812 N was melted at 75 °C on a magnetic stirrer (700 rpm). The aqueous phase (50 mL) containing 500 mg Tween 20 was heated to the same temperature and added dropwise to the lipid one. Afterwards, the hot pre-emulsion was stirred for 10 min at 1000 rpm and then sonicated at 45% amplitude with the probe sonicator Bandelin SonuPuls (Bandelin Sonoplus HD3100, Bandelin Electronics, Berlin, Germany) for 4 min (4303 kJ energy). The nanoparticles were obtained by solidification in ice bath. The empty nanoparticles were coded as NLC. The berberine (50 mg) was loaded by dissolving in the molten lipids and the thus obtained nanoparticles were labeled as NLC-B. Three batches of each formulation were prepared for comparison purposes.

#### 2.2.2. Nanoparticles Characterization

The hydrodynamic diameter, polydispersity index, and zeta potential of the NLC and NLC-B were evaluated with Dynamic Light Scattering analysis (DLS) using Zeta-master (Malvern Instruments, Malvern, UK) with DTS0070 cuvette. All samples were measured as aqueous dispersions immediately after preparation after dilution 1:100 at a scattering angle of a 90° in triplicate similar to previous NLC studies [36].

Transform Infrared Spectroscopy (Thermo Scientific Nicolet, Waltham, MA, USA) was used to collect the spectra of the initial components and the prepared NLCs in the range of 4000–400 cm^−1^ and with a resolution of 2 cm^−1^.

The size and shape of the NLCs was evaluated by transmission electron microscopy (JEOL JEM 2100 h STEM JEOL, Tokyo, Japan) at 200 kV and point resolution = 0.23 nm. A droplet of the aqueous dispersion was deposited on a copper grid and let to air dry prior to observation [37,38].

The solid state analysis was performed in the solid state of the freeze-dried samples. The freeze-drying was performed with the help of a semi-industrial freeze-drier (Martin Christ, Gefriertrocknunganlagen GmbH, Osterode, Germany). The process was carried in two pahses (first drying at −110 °C and 0.125 mbar for 72 h, followed by a second drying at −110 °C and 0.05 mbar for 6 h). Thermogravimetric analyses (TGAs) were performed on a Perkin Elmer TGA 4000 (Waltham, MA, USA). Measurements were run at a heating rate of 10 °C·.min^−1^ under a nitrogen flow rate of 20 mL·min^−1^, to avoid any thermo-oxidative degradation. Instrument control, data acquisition, and data processing were performed by Pyris software (v.11.0.0.0449). The ±1 °C accuracy on the degradation temperatures determined from the derivatives of the weight losses versus temperature curves was established (Supplementary Materials). The melting and crystallization of Precirol^®^ 5 ATO (Prec), berberine (BRB), the physical mixture of Precirol^®^ 5 ATO, Tween 20, and Mygliol^®^ 812 N without berberin (PM), the physical mixture of Precirol^®^ 5 ATO, Tween 20, and Mygliol^®^ 812 N with berberine (PMB), empty (NLC), and berberine (NLC-B)-loaded nanoparticles were studied by differential scanning calorimetry (DSC Perkin Elmer 8500 with a refrigerated cooling accessory Intercooler) in the −70 to 200 °C temperature range, at a heating rate of 10 °C.min^−1^ under nitrogen flow (50 mL·min^−1^). The freeze-dried solid-state samples (6.0 ± 0.1 mg) were placed in aluminum DSC pans. The pans were hermetically sealed and the DSC study was initiated by program heating from −70 to 200 °C at a rate of 20 °C·min^−1^ and then quenching to room temperature. The control of the device, data collection, and processing were performed with the specialized Pyris software v.10.1.0.0412. The melting temperature (T_m_) was defined as the temperature maximum of the melting endotherm of the first heating run.

The amount of loaded berberine was determined with a two-step procedure. A certain amount of the nanodispersion was filtered by 0.45 µm (filter). Afterwards, the filter was washed with methanol and the amount of the free undissolved berberine retained by the filter was spectrophotometrically determined. In order to determine the dissolved free berberine, the filtrate was then centrifuged using a separatory centrifuge tube (Vivaspin 500, VWR International, Darmstadt, Germany) at 6000 rpm for 15 min. The aqueous phase was analyzed spectrophotometrically, and the amount of free berberine (BRB_free_) was calculated using the standard curve. The loading efficiency (EE%) was calculated using the following Equation (1) based on the total charged amount of BRB (BRB_total_):

(1) $$\mathrm{EE}\%=\frac{{BBR}_{total}-{BBR}_{free}}{{BBR}_{total}}x100$$

Further, the loading capacity was calculated based on Equation (2):

(2) $$LC\%=\frac{{BBR}_{total}-{BBR}_{free}}{{Nanoparticles}_{weight}}\times 100$$

The nanoparticles’ weight was determined by collecting the nanoparticles separated by the centrifuge tube, air-drying them at room temperature and accurately weighing the amount (Mettler AJ150, Mettler Toledo AG, Urdorf, Switzerland).

The In Vitro release profile was determined by a dialysis bag method, which is considered a ‘method of choice’ for nanoparticulates [39]. It followed a previously described procedure [40,41] using a shaking water bath at 37 ± 0.5 °C and 100 rpm horizontal agitation. The test sample (NLC-B dispersion or aqueous free BRB dispersion), corresponding to approximately 2 mg berberine, was placed in a Spectra/Por^®^ dialysis membrane from regenerated cellulose (VWR International GmbH, Darmstadt, Germany) with a sufficient cut-off limit (10,000 Da) to allow for unrestricted drug diffusion [42]. Phosphate-buffered saline (PBS) with pH 7.4 was used as the release medium. Aliquots of 1.5 mL were taken at predetermined intervals and the volume of the medium was kept constant by adding 1.5 mL of fresh PBS. The amount of berberine released was measured spectrophotometrically at 430 nm. All tests were conducted in triplicate. The release profiles were fitted for various kinetics equations, namely zero-, first-order, Higuchi, and Korsmeyer–Peppas models, with the help of the free plug-in for Excel DDsolver.

#### 2.2.3. Cell Lines

The CaCo-2 cells (human colorectal adenocarcinoma cell line), HuCC-T1 cells (human cholangiocellular carcinoma cell line), L929 cells (fibroblast-like cell line), and H9c2 cells (rat cardiomyoblast cell line) were obtained from the European Collection of Authenticated Cell Cultures (ECACC, Salisbury, UK). L5178y cells (mouse lymphoma cell line) were provided by Dr. M. M. Gottesman (National Cancer Institute, Bethesda, MD, USA). The cells were cultured under strict aseptic conditions in McCoy′s 5A Medium or Dulbecco’s Modified Eagle Medium (DMEM) with either high or low glucose concentrations, supplemented with 10% Fetal Bovine Serum (FBS) and 2 mM l-glutamine solution. They were maintained at a constant temperature of 37 °C in a humidified atmosphere with 5% CO_2_. The culture medium was refreshed every two days to ensure optimal growth conditions.

#### 2.2.4. Cell Viability Assays

Dispersions of berberine (BRB), berberine-loaded nanostructured lipid carriers (NLC-B), and empty nanostructured lipid carriers were prepared. The 96-well plates containing the cell lines were treated with a series of dilutions of the aforementioned test dispersions. The concentrations of BRB tested were in the range of 0.1–200 µM, while the concentrations of NLC tested were in the range of 1–2000 µg/mL. Each concentration of the test dispersions was added to six wells on each plate, with control wells containing only culture medium. The plates were then incubated at 37 °C in a humidified atmosphere of 5% CO_2_ for 72 h for CaCo-2 cells and HuCC-T1 and 24 h for L5178y, L929, and H9c2 cells.

The cytotoxicity and safety profiling of the three types of test dispersions were assessed using the MTT (3-(4,5-dimethylthiazol-2-yl)-2,5-diphenyltetrazolium bromide) colorimetric assay [43] or the resazurin assay [44].

For adherent cultures, we applied the MTT assay. Briefly, MTT was dissolved in phosphate-buffered saline at a concentration of 10 mg/mL. Following treatment with the test solutions and aspiration of the culture medium, 100 µL of the MTT solution was added to each well of the treated cells. The plates were then incubated for 3 h at 37 °C. After incubation, the medium was aspirated, and 100 µL of dimethyl sulfoxide (DMSO) was added to each well to dissolve the purple formazan product. The absorbance of the resulting solutions was measured at a wavelength of 570 nm using a Synergy 2 microplate reader (BioTek Instruments, Inc., Highland Park, Winooski, VT, USA). This method enabled the quantification of the cytotoxic effects of empty NLC, BRB, NLC-B on the five selected cell lines.

For suspension cultures, we applied the resazurin assay. Briefly, resazurin was added to wells at a final concentration of 0.001 mg/mL and after 2 h of incubation at 37 °C, fluorescence was measured at excitation wavelength of 540 nm and emission wavelength of 620 nm in a Synergy 2 plate reader (BioTek Instruments, Inc., Highland Park, Winooski, VT, USA).

#### 2.2.5. Comet Assay and Image Analysis

The alkaline comet assay was applied in order to identify and quantify genotoxic damage [45]. L5178y cells were seeded in 96-well plates at 8 × 10^4^ cells per well and treated with NLC (175, 350 or 700 µg/mL), culture medium for negative controls or 175 µM H_2_O_2_ for 30 min at 37 °C for positive controls. Microscope slides were coated with a solution of 1% normal melting point agarose. After 24 h, cells re-suspended in PBS and mixed with 0.7% low-melting-point agarose in a 1:1 ratio were applied to the slides, covered with coverslips, and allowed to solidify at 4 °C.

Slides were lysed in a buffer containing 2.5 M NaCl, 100 mM EDTA, and 10 mM Tris (pH 10) for 90 min at 4 °C. DNA unwinding was performed by immersing the slides in cold electrophoresis buffer (300 mM NaOH, 1 mM Na_2_EDTA, pH 13) for 30 min at 4 °C. Electrophoresis was conducted in the dark at 10 V and 300 mA for 20 min. Slides were neutralized in 0.4 M Tris buffer (pH 7.5), rinsed with distilled water, and stained with SYBR Green. Slides were allowed to dry at room temperature prior to analysis.

DNA migration patterns were visualized by fluorescent microscopy in the dark to minimize additional DNA damage from UV light. Quantitative data on the characteristics of the comets were obtained using ImageJ 1.54d (Wayne Rasband and contributors National Institute of Health, Bathesda, MD, USA) [46] software and the OpenComet plug-in [47]. The Olive moment of the comets was used as genotoxicity parameter as it accounts for both the amount of damaged DNA and its distribution.

#### 2.2.6. Experimental Animals

The study was conducted using five male white Wistar rats. The animals were supplied by the National Breeding Center of the Bulgarian Academy of Sciences, located in Slivnitsa, Bulgaria. All experiments were performed in compliance with Regulation No. 15 on the protection and humane treatment of experimental animals (SG No. 17, 2006) and in accordance with European standards for working with experimental animals. The study protocol was approved by the Bulgarian Food Safety Agency under permit No. 323, valid until 22 December 2026.

#### 2.2.7. Isolation of Liver Microsomes and FeSO_4_/Ascorbic Acid-Induced Lipid Peroxidation in Isolated Liver Microsomes

Immediately before the experiment, rats are anaesthetized with Pentobatbital Na (0.2 mL/100 g i.p.). Livers were perfused with 1.15% KCl and homogenized in four volumes of ice-cold 0.1 M potassium phosphate buffer (pH 7.4). The homogenate was centrifuged at 9000× *g* for 30 min at 4 °C to obtain the post-mitochondrial fraction (S9), which was further centrifuged at 105,000× *g* for 60 min at 4 °C. The resulting microsomal pellets were re-suspended in 0.1 M potassium phosphate buffer (pH 7.4) containing 20% glycerol. Liver microsome aliquots were stored at −70 °C until analysis [48]. Microsomal protein content was measured using the method of Lowry et al. [49], with bovine serum albumin as the standard.

Isolated microsomes were pre-incubated with NLC, BRB, and NLC-B for 60 min at 37 °C. Lipid peroxidation (LPO) was initiated by adding 20 µM iron sulfate and 0.5 mM ascorbic acid to the microsomes at 37 °C. After 20 min, the reaction was terminated by adding a mixture of 25% TCA and 0.67% TBA. The malondialdehyde (MDA) levels were subsequently quantified to assess LPO [50].

#### 2.2.8. Statistical Analysis

Statistical analyses were conducted using GraphPad Prism version 8 (GraphPad Software, Boston, MA, USA). Data were evaluated from three independent experiments. Group means were compared using one-way analysis of variance (ANOVA), with Dunnett’s post-test applied for parametric data and the Kruskal–Wallis test with Dunn’s post-test for nonparametric data. A significance threshold of *p* < 0.05 was used to determine statistical significance in all comparisons. Outliers were identified and removed using iterative Grubbs’ test, ensuring the accuracy and reliability of the analysis. Statistical analysis was performed to compare the different groups, NLC-B and BRB. Statistical significance determined using the Holm–Sidak method, with alpha = 0.05. Each row was analyzed individually, without assuming a consistent SD. Number of *t* tests: 9.

## 3. Results

### 3.1. Nanoparticle Preparation and Their Physicochemical and Biopharmaceutical Characterization

The investigation of BRB solubility in the tested excipients showed maximal values for Precirol^®^ 5 ATO, Tween 20 and Mygliol^®^ 812 N, as shown in Table 1. Therefore, they were applied for the preparation of the empty and drug-loaded NLCs.

**Figure 2:**
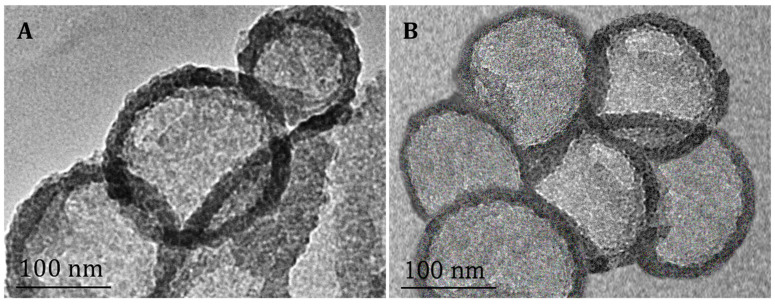
Transmission electron microscopy images of empty NLC (**A**) and BRB-loaded (NLC-B) nanoparticles (**B**) at 50 k magnification.

The melt-emulsification and ultrasonication technique successfully yielded empty (NLC) and berberine-loaded (NLC-B) nanoparticles. Their average hydrodynamic size, polydispersity index, and negative zeta potential are shown in Table 2, and the z-average size reported by intensity, volume, and number are presented in Supplementary Materials Figure S2. The nanoparticles’ morphology characterized by TEM is visualized on Figure 2A,B.

**Figure 3:**
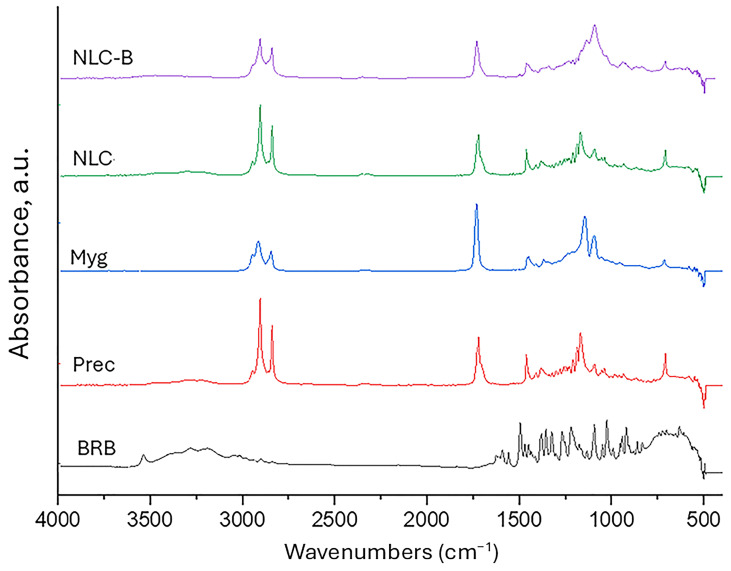
FTIR spectra of the initial components, empty NLC, and berberine-loaded NLC-B (Myg—Mygliol^®^ 812N; Prec—Precirol^®^ 5 ATO).

Berberine was successfully encapsulated (EE% = 97.7 ± 2.3% and LC% = 3.46 ± 1.7%) as evident from the presented IR spectra (Figure 3). In the spectrum of BRB, peaks are evident at 3057 cm^−1^ for the quaternary ammonium group, at 2910 cm^−1^ for C-H stretching, at 2855 cm^−1^ for -OCH_3_ (methoxyl group), at 1627 cm^−1^ for the C-N band, and at 1601cm^−1^ for the stretching vibration band of C=N^+^ (quaternary iminium ion) [51]. The peak at 1507 cm^−1^ was related to the vibration of the aromatic C-C bond. The ring deformation and CH in-plane bending are present in the peak at 1110 cm^−1^. The band at 1039 cm^−1^ characterizes C-H vibrations in the ring, as well as the peaks at 3292 cm^−1^ and 3546 cm^−1^. Similar data are reported by Bashmakova et al. [52].

In the spectrum of Precirol^®^ 5 ATO, the characteristic peaks of C-C stretching are presented at 1470 cm^−1^. The peak at 1730 cm^−1^ corresponds to the C=O stretching, and the bands at 2914 cm^−1^ and 2850 cm^−1^ correspond to the C-H stretching. Similar results were obtained by other authors [53,54].

The Mygliol^®^ 812 N spectrum shows characteristic peaks of C=O stretching at 1738 cm^−1^, of C-H stretching at 2846 cm^−1^ and 2926 cm^−1^, and C-C stretching is presented at 1462 cm^−1^.

In the spectrum of NCL, both lipids are presented, and no chemical (i.e., only hydrophilic–hydrophobic) interactions between them are observed. The FTIR spectra of the individual components, empty NLC, and berberine-loaded NLC (NLC-B) are included in the Supplementary Materials.

**Figure 4:**
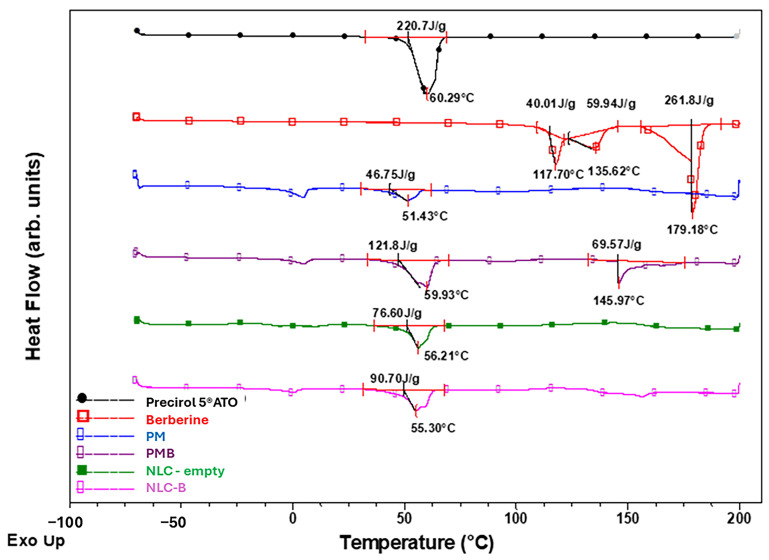
DSC thermograms of Precirol^®^ 5 ATO, berberine, physical mixture (PM), physical mixture with berberine (PMB), empty (NLC), and berberine (NLC-B)-loaded nanoparticles.

The contributions of the lipids to the melting behavior and crystalline state of the physical mixture of Precirol^®^ 5 ATO, Tween 20z and Mygliol^®^ 812 N(PM), the physical mixture of Precirol^®^ 5 ATO, Tween 20, and Mygliol^®^ 812 N with berberine (PMB), empty (NLC), and berberine (NLC-B)-loaded nanoparticles were investigated by DSC. Thermograms are presented in Figure 4 and data are summarized in Table S1 in the Supplementary Materials.

**Figure 5:**
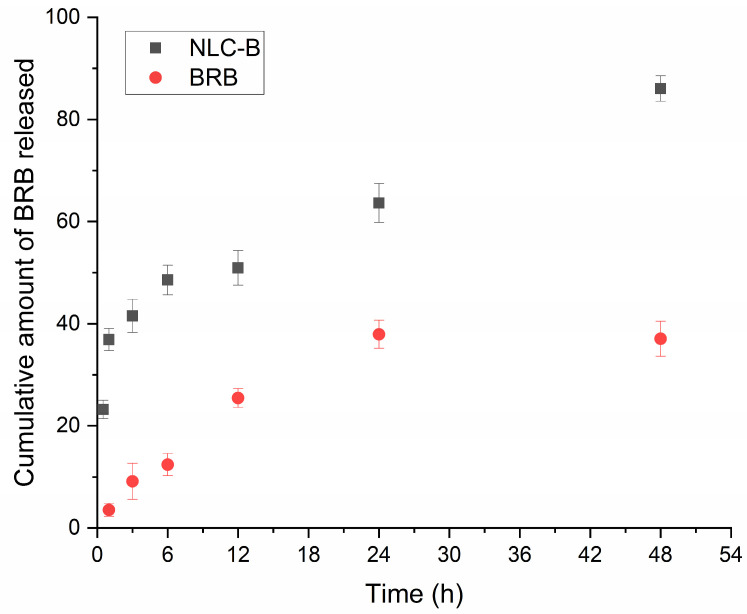
Release profile of free BRB and NLC-B in PBS with pH = 7.4 (mean ± SD; n = 3).

The In Vitro release characteristics of the free drug and the loaded NLCs in PBS are presented in Figure 5. The nanoparticle loading results in an improved dissolution profile over time (released amount 86.0 ± 2.49%), while the free drug is not completely dissolved (37.05 ± 3.43%) after 48 h. A sustained profile is also observed for the NLC-B following zero-order kinetics, having the highest correlation coefficient (R^2^ = 0.9402).

### 3.2. Cell Experiments

#### 3.2.1. In Vitro Cytotoxicity Effects of NLC, BRB, and NLC-B on CaCo-2 and HuCC-T1 Cell Lines

**Figure 6:**
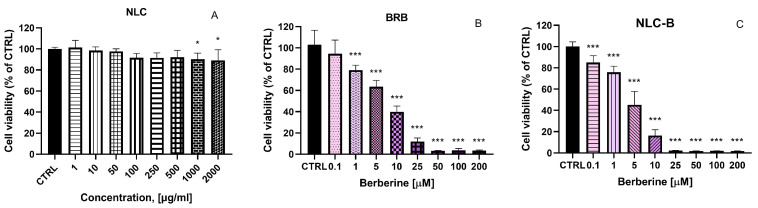
In Vitro evaluation of the cytotoxicity of (**A**) empty NLC (1, 10, 50, 100, 250, 500, 1000, 2000 µg/mL), (**B**) BRB, and (**C**) NLC-B (0.1, 1, 5, 10, 25, 50, 100, 200 µM) on CaCo-2, 72 h after treatment, by means of MTT assay; * *p* < 0.05, *** *p* < 0.001. Statistical analysis was performed using ANOVA with a post-test of Dunnett. Values are presented as percentages of untreated cells and expressed as average values ± SEM (n = 6).

The precise mechanisms underlying berberine’s therapeutic action in colorectal cancer treatment remain insufficiently understood. Therefore, we performed an initial investigation into the effects of BRB and NLC-B on two tumor cell lines—CaCo-2 (human colorectal adenocarcinoma) and HuCC-T1 (human cholangiocarcinoma cell line). An evaluation of the toxicity of empty NLC, BRB, and NLC-B was conducted by treating the CaCo-2 cells with varying concentrations. Cell viability was evaluated via the MTT assay as a means of assessing cell metabolic activity 72 h after treatment (Figure 6). No cytotoxicity of the empty NLC was observed (Figure 6A). In contrast, free BRB and NLC-B showed a statistically significant and concentration-dependent decrease in cell viability in concentrations of 1 µM and higher (Figure 6B,C). It is important to highlight that the toxicity of the berberine-loaded nanoparticle NLC-B is higher compared to the free substance, as clearly demonstrated by the IC_50_ values obtained: IC_50_ (NLC-B) = 3.35 µM, IC_50_ (BRB) = 8.42 µM. Higher cytotoxicity of NLC-B compared to BRB was observed at concentrations of 1, 10, 25, 50, and 200 µM, as determined using the Holm–Sidak method with alpha = 0.05. This observation suggests that the encapsulation of berberine within nanoparticles may alter its interaction with cellular systems, potentially enhancing its cytotoxic effects. It could be also associated with the improved drug solubility as demonstrated in the In Vitro release study.

**Figure 7:**
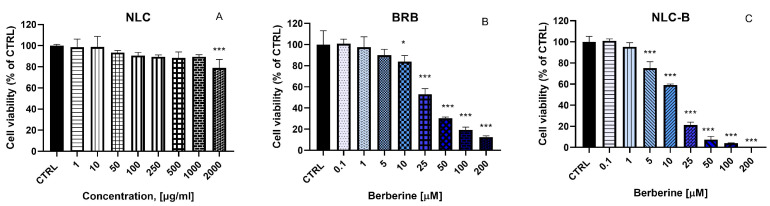
In Vitro evaluation of the cytotoxicity of (**A**) empty NLC (1, 10, 50, 100, 250, 500, 1000, 2000 µg/mL), (**B**) BRB, and (**C**) NLC-B (0.1, 1, 5, 10, 25, 50, 100, 200 µM) on HuCC-T1 72 h after treatment, by means of MTT assay; * *p* < 0.05, *** *p* < 0.001. Statistical analysis was performed using ANOVA with a post-test of Dunnett. Values are presented as percentage of untreated cells and expressed as average values ± SEM (n = 6).

In the subsequent stage of the study, we evaluated the effects of NLC, BRB, and NLC-B on the HuCC-T1 cell line (Figure 7). Within the tested concentration range (1–2000 µg/mL), NLC demonstrated no detectable cytotoxic effects on cells after 72 h of treatment. In contrast, free berberine (0.1 and 200 µM) showed a significant reduction in cell viability at concentrations above 10 µM. Similarly, berberine encapsulated within lipid nanoparticles exhibited cytotoxic effects at even lower concentrations, with significant reductions in cell viability observed at concentrations above 5 µM. This enhanced cytotoxic activity highlights the impact of nanoparticle-based delivery systems in increasing the efficacy of therapeutic agents. The results indicate a substantial increase in the cytotoxic potency of berberine when incorporated into the lipid nanoparticle system compared to its free form. This observation is further corroborated by the comparison of IC_50_ values, where the IC_50_ for the lipid nanoparticle-loaded berberine (NLC-B) was calculated as 11.86 µM, markedly lower than the IC_50_ for free berberine, which was 24.66 µM. Higher toxicity of NLC-B compared to BRB was observed at concentrations of 5, 10, 25, 50, 100, and 200 µM, as determined using the Holm–Sidak method with α = 0.05. These findings might be considered to be related to the improved cellular uptake of encapsulated berberine and the prolonged release from the nanoparticulate system.

#### 3.2.2. In Vitro Safety Evaluation of NLC, BRB, and NLC-B

##### Toxicity Assessment on L929 and H9c2 Cell Lines

**Figure 8:**
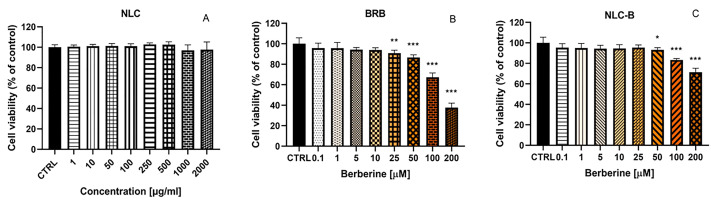
In Vitro evaluation of the cytotoxicity of empty (**A**) NLC (1, 10, 50, 100, 250, 500, 1000, 2000 µg/mL), (**B**) BRB, and (**C**) NLC-B (0.1, 1, 5, 10, 25, 50, 100, 200 µM) on L929 24 h after treatment, by means of MTT assay; * *p* < 0.05, ** *p* < 0.01, *** *p* > 0.001. Statistical analysis was performed using ANOVA with a post-test of Dunnett. Values are presented as percentage of untreated cells and expressed as average values ± SD (n = 6).

The well-established experimental models, such as isolated subcellular fractions and immortalized cell lines, are widely used in nanotoxicity evaluations due to their reliability and relevance [55]. The next study aimed to determine the potential cytotoxic effects associated with the treatments and to determine their compatibility with cell viability on L929 (fibroblast cells) and H9c2 (cardiomyocytes) cell lines. L929 cells are particularly suitable for these types of assessments because of their sensitivity to a wide range of chemical and biological agents, providing a reliable measure of cellular response to test substances. These cells play a critical role in preclinical evaluations of novel therapeutic agents and nanomaterials [56]. The toxicity of empty NLC, BRB, and NLC-B was evaluated by treating L929 cells with a series of increasing sample concentrations (Figure 8). Cell viability was assessed 24 h after treatment using the MTT assay. The results revealed that the empty NLC did not exhibit detectable cytotoxicity (Figure 8A). We found that the empty NLC carrier is a safe and biocompatible system, which does not adversely impact the metabolic activity of L929 cells. This finding supports its potential use in drug delivery applications.

While BRB and NLC-B did not induce cytotoxic effects at lower concentrations, their impact on cell viability became significant at concentrations of 100 µM and above (Figure 8B,C). This observation suggests that BRB contributes to the observed cytotoxic effects, and these effects are retained when BRB is encapsulated in the NLC system. However, a comparison of the cytotoxic profiles between BRB and NLC-B indicates a potential mitigation of toxicity of encapsulated BRB. Lower cytotoxicity of NLC-B compared to BRB was observed at concentrations of 50, 100, and 200 µM, as determined using the Holm–Sidak method with α = 0.05. Thus, NLC-B appears to reduce the cytotoxicity of BRB at the highest tested concentration (200 µM) with NLC-B showing 62% cell viability compared to 29% for BRB alone. The results suggest that encapsulation into NLC may offer a safer delivery mechanism for BRB by attenuating its cytotoxic effects on healthy, non-tumor cells, especially at high concentrations. This highlights the potential utility of NLC for reducing the adverse effects of potent therapeutic agents like BRB.

**Figure 9:**
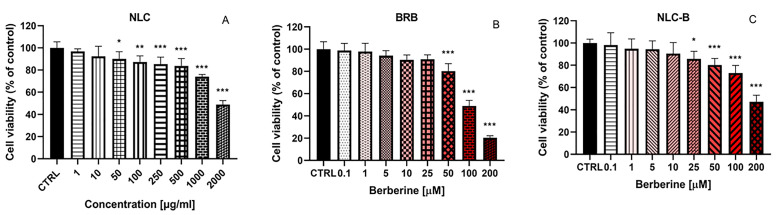
In Vitro evaluation of the cytotoxicity of empty (**A**) NLC (1, 10, 50, 100, 250, 500, 1000, 2000 µg/mL), (**B**) BRB, and (**C**) NLC-B (0.1, 1, 5, 10, 25, 50, 100, 200 µM) on H9c2 cells 24 h after treatment, by means of MTT assay; * *p* < 0.05, ** *p* < 0.01, *** *p* > 0.001. Statistical analysis was performed using ANOVA with a post-test of Dunnett. Values are presented as percentage of untreated cells and expressed as average values ± SEM (n = 6).

H9c2 cell line was selected as a cardiomyocyte model to evaluate the cardiotoxicity profile of empty NLC, BRB, and NLC-B [57]. This cell model was chosen due to its relevance in studying cardiac cell responses to various treatments, including potential toxic effects. The toxicity evaluation was conducted by exposing H9c2 cells to a range of concentrations and assessing cell viability 24 h post-treatment. The results, illustrated in Figure 9, reveal distinct toxicity profiles. In concentration range from 1 to 1000 µg/mL the empty NLC did not exhibit any detectable adverse effects on cell viability, indicating their biocompatibility. Moderate cytotoxic effects were observed only at the highest NLC concentrations, specifically 1000 µg/mL and 2000 µg/mL.

For BRB, a marked reduction in cell viability was observed at concentrations of 100 µM and above (Figure 9B). This indicates a dose-dependent cytotoxicity, consistent with the known pharmacological properties of BRB. The inherent BRB toxicity at elevated doses poses a challenge for its use in therapeutic applications, particularly for sensitive healthy cells such as cardiomyocytes. NLC-B also showed cytotoxicity effects, especially at concentrations of 100 µM and above (Figure 9C). However, a significant finding was that the encapsulation of BRB into NLC markedly reduced its toxicity, compared to BRB alone. At the highest concentration (200 µM), NLC-B demonstrated significantly higher cell viability compared to BRB, with 47% cell viability versus 20%, respectively. This reduction in cytotoxic effects underscores the protective role to healthy cells of the NLC, likely due to its ability for sustained active substance release, thereby mitigating its immediate toxic response to the cells.

##### In Vitro Genotoxicity Assay of NLC

**Figure 10:**
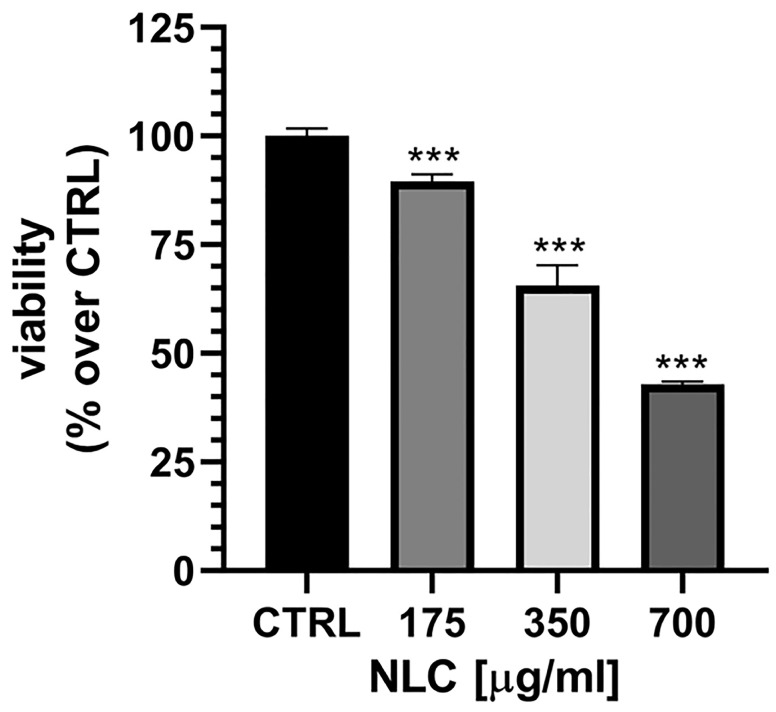
Cell viability of L5178y cells, treated with NLC at 175, 350 and 700 µg/mL, normalized vs. controls (CTRL). Mean ± SD. *** *p* < 0.001, one-way ANOVA with Dunnet’s post-test.

Initial cell viability studies were carried out to determine an appropriate concentration for genotoxicity studies, at which the test substance exerts significant, but not extensive cellular damage. L5178y cells were treated with 175, 350 or 700 µg/mL NLC and in all of the treatment groups we observed a different degree of cytotoxic effects (Figure 10). Those effects were weak at the treatment concentration 175 µg/mL and pronounced at 700 µg/mL. Among the tested concentrations, the effects were moderate at 350 µg/mL and this concentration was chosen for the comet assay.

**Figure 11:**
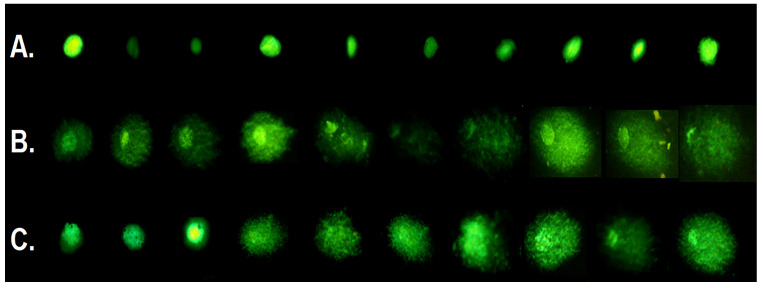
Representative fluorescent micrographs of L5178y cells, treated with NLC (**C**), negative (**A**) or positive (**B**) control after being subjected to gel electrophoresis and stained with sybr green.

To evaluate the genotoxic potential of NLC, we subjected treated L5178y cells to the comet assay. After membrane lysis, gel electrophoresis of DNA fragments and DNA staining, the treated cells formed the characteristic comets. Figure 11 shows representative images of micrographs after the comet assay. Due to the low amount of DNA fragmentation, control cells’ micrographs (Figure 11A) are compact and less spread. Peroxide control, peroxide-treated cells, on the other hand, formed well pronounced comets in most of the micrographs (Figure 11B). Upon treatment with 350 µg/mL, the comet assay reveals the presence of cells with different sensitivity to genotoxic damage, which is visible from the presence of fractions with well pronounced, moderately pronounced comets, as well as cells, which do not form comets (Figure 11C).

**Figure 12:**
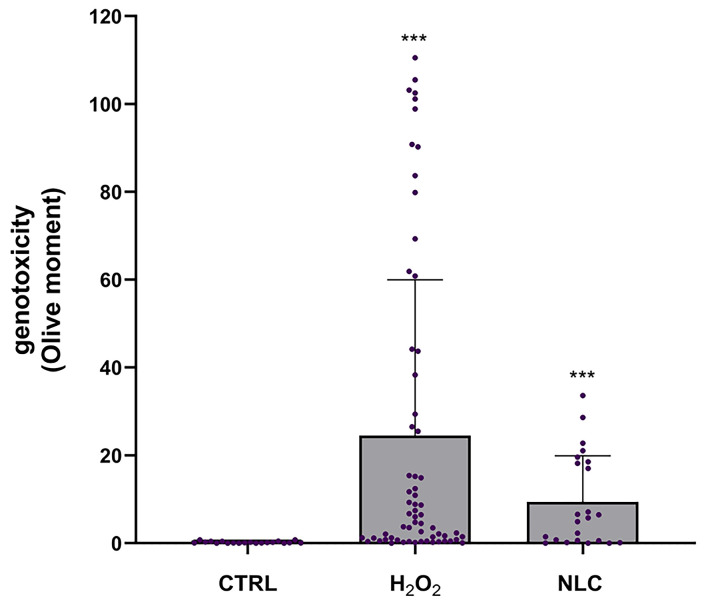
Olive moments of L5178y cells, subjected to the comet assay after treatment with NLC at 350 µg/mL, negative (CTRL) or positive (H_2_O_2_) controls. Mean ± SD; *** *p* < 0.001 vs. CTRL. Kruskal–Wallis test with Dun’s post-test.

In order to better understand the differences between comets, we applied image analysis for extracting quantitative information from the micrographs. The obtained results confirm that NLC treatment, as well as positive, hydrogen peroxide-treated controls result in statistically significant development of more pronounced comets, compared to negative controls (Figure 12). The superimposed dot plots show that there are fractions of cells with different sensitivity to genotoxic damage in both NLC and positive control-treatment. However, with NLC treatment the most pronounced comets have several fold lower Olive moment values, compared to the most pronounced comets of hydrogen peroxide-treated cells.

##### Effects of NLC, BRB, and NLC-B on Isolated Rat Liver Microsomes

**Figure 13:**
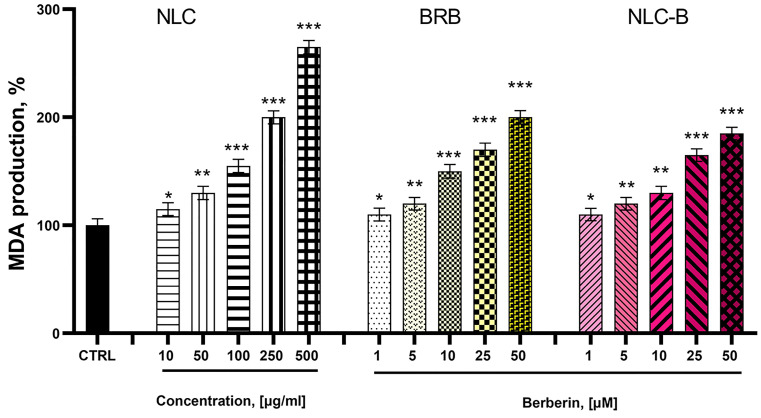
Effects of NLC, BRB, and NLC-B on MDA production in isolated rat liver microsomes. Data are presented as means from three independent experiments ± SD (n = 3). * *p* < 0.05; ** *p* < 0.01. *** *p* < 0.001 vs. control (non-treated microsomes).

Rat liver microsomes represent one of the most widely used In Vitro models in toxicity evaluation studies. Liver microsomes are subcellular fractions derived from the endoplasmic reticulum of hepatic cells, prepared through liver homogenization [58] and differential centrifugation [59]. Rat liver microsomes are a rich source of drug-metabolizing enzymes, including cytochrome P450s, flavin monooxygenases, carboxyl esterases, epoxide hydrolases, and UDP-glucuronyl transferases. As such, they serve as a valuable In Vitro system for investigating the metabolic fate of xenobiotics, including nanoscale drug delivery systems [60]. When administered alone, all samples (NLC, BRB, and NLC-B) exhibited a statistically significant, concentration-dependent pro-oxidant effect compared to the control (non-treated liver microsomes) (Figure 13). For free BRB, a concentration of 1 µM increased MDA production statistically significantly by 10%, 5 µM by 20%, 10 µM by 50%, 25 µM by 70%, and 50 µM by 100% compared to the control. For NLC-B, a concentration of 1 µM increased MDA production statistically significantly by 10%, 5 µM by 20%, 10 µM by 30%, 25 µM by 65%, and 50 µM by 85% compared to the control. Notably, at higher concentrations, berberine-loaded nanoparticles NLC-B demonstrated lower toxicity compared to free BRB.

## 4. Discussion

Tumors of the digestive tract are among the most common cancers, with colorectal carcinoma being the second most prevalent in Europe [61]. Natural substances with proven antiproliferative properties, such as berberine, are valuable additives taken together with conventional antitumor drugs [62]. Berberine possesses various pharmacological properties, including antioxidant and antitumor activities [63]. Due to its affinity for the liver, berberine can be used as a targeting ligand for bile ducts.

In this study, we developed nanostructured lipid carriers (NLCs) to address their pharmacokinetic challenges and to evaluate their In Vitro antitumor and safety effects.

Nanostructured lipid carriers with and without berberine were successfully prepared by the melt-emulsification and ultrasonication technique. The particle sizes are relatively small and thus suitable for the parenteral or oral route of administration. Similar results have been published before [34]. The observed size falls in the suitable range of 100–200 nm in diameter to expect an enhanced permeation and retention (EPR) effect in solid tumors [64]. This could be associated with an improved therapeutic outcome. The high entrapment efficiency observed in the current study could be due to the high solubility of BRB in the chosen excipients, which is in accordance with previous data [34,65]. The loading capacity is similar to previously prepared NLCs with berberine, investigated for their application in ulcerative colitis [36]. The negative zeta potential of the nanocarriers provides them with sufficient physical stability, as values higher than |30 mV| are considered optimal in this regard [66]. This negative potential is most likely due to the presence of free fatty acids within the lipid matrix and their carboxyl group dissociation [67]. The presence of the amine group in berberine’s structure is associated with a decrease in the zeta potential of the loaded nanocarrier, but still remains negative, as shown in other studies [68]. The negative value additionally favors potential prolonged circulation [64].

Regarding the In Vitro release behavior, it is evident that the loading of BRB in the NLC nanocarrier leads to the improvement of its dissolution in the physiological pH. This is probably associated with the increased surface area of the nanoparticles, together with the presence of the surfactant, namely Tween 20, which improves the wettability of the NLCs. Simultaneously, a prolonged release is observed, which could contribute to the extended effect of the drug on the targeted tissue. Similar results were reported in the literature, even though higher amounts were released within 12 h [69]. The difference could be attributed to the different formulation composition or the application of the dialysis membrane in our research, which could affect drug diffusion. The proposed nanocarrier exhibited zero-order release, which is considered advantageous for parenteral formulations as it guarantees the constant release of berberine. This would guarantee a concentration-independent release and avoiding a sudden increase in the available drug in the blood. Such is considered a prerequisite for a potential better safety profile [70,71].

The FTIR spectra confirmed the successful encapsulation of berberine in the NLC system. Key peaks corresponding to berberine’s quaternary iminium ion and aromatic C-C bonds were absent in the NLC-B spectrum, indicating its integration into the lipid matrix. Additionally, the structural integrity of the lipid components was maintained without significant interactions between them. These findings corroborate the high encapsulation efficiency and enhanced dissolution characteristics of berberine-loaded NLCs.

The performed DSC study of Prec, pure BRB, empty (NLC), berberine-loaded lipid nanoparticles (NLC-B), and physical mixtures reveal that BRB itself showed a very high melting temperature in the interval between 118 and 178 °C, while the lipids were characterized by a single endotherm in the range of 50 ÷ 60 °C. Representative DSC curves together with the corresponding characterization data are shown in Figure 4. The physical mixture (PMB sample) has one low melting point around 60 °C, corresponding to the lipid part, and a second endothermic peak around 146 °C due to BRB. Based on these observations, we could suggest that BRB in the PMB is crystalline. The DSC endotherms of the berberine-loaded lipid nanoparticles (NLC-B sample) showed one single melting endotherm at about 56 °C near the melting point of pure Precirol^®^ 5 ATO, and not surprisingly, the peak corresponding to the drug in the interval of 118 and 178 °C was not observed. On this basis, we could suggest that the berberine is encapsulated in the hydrophobic compartment of the prepared lipid nanoparticles (NLC-Bs) and is in the amorphous or non-crystalline form. This is a particularly important observation, suggesting that the results are consistent with the data obtained from DLS and FTIR analysis and can further explain the improved solubility In Vitro.

The choice of the In Vitro design for the biological characterization was aimed at investigating the effects of compounds in a panel of cell lines and a subcellular fraction from primary cells with phenotypes, characteristic not only for gastrointestinal tumors (HuCC-T1 and Caco-2), but also for off-target tissues such as cardiac (H9c2), connective (L929) tissue, and rat liver microsomes. Moreover, to gain insight into the presence of more subtle toxic manifestations, genotoxicity in the L5178y cell line was studied. This is a widely adopted choice for genotoxicity studies [72].

The cytotoxicity studies of two tumor-derived cell lines, HuCC-T1 (human cholangiocarcinoma) and CaCo-2 (colorectal adenocarcinoma), demonstrated the antiproliferative potential of free BRB and encapsulated NLC-B, with the latter exhibiting enhanced cytotoxic effects. The IC_50_ values for NLC-B (3.35 µM for CaCo-2 and 11.86 µM for HuCC-T1) were significantly lower than those for free BRB, indicating the increased potency of encapsulated berberine. This enhancement is likely due to the nanoparticle-mediated delivery, which could improve the cellular uptake and sustained release of BRB, as well as its interaction with tumor cells. The observed reduction in HuCC-T1 and CaCo-2 cell viability is dose-dependent and consistent with the known antitumor mechanisms of BRB, such as its ability to induce apoptosis and to disrupt mitochondrial function [73]. This finding aligns with the known cytotoxic properties of berberine, which are dose-dependent and reflect its potent bioactivity. Wei He et al. demonstrated that berberine treatment of human cholangiocarcinoma QBC939 cells reduced cell viability and induced dose-dependent cell death, accompanied by G1 cell cycle arrest. Furthermore, berberine treatment (10, 40, 80 µM for 48 h) significantly increased apoptosis, with elevated pro-apoptotic Bax levels and decreased anti-apoptotic proteins Bcl-2 and Bcl-xL [74]. The enhanced effect of NLC-B compared to free BRB could be attributed to the nanocarrier’s ability to overcome limitations of poor solubility and low permeability, typically associated with the physicochemical properties of free BRB. Similar findings have been reported by other researchers. Wang et al. developed berberine-loaded nanostructured lipid carriers (NLC-Bs) using hot melting and high-pressure homogenization. The resulting particles measured 189.3 nm in size and had a negative zeta potential of −19.3 mV. MTT assays revealed that NLC-B significantly inhibited H22 cell proliferation, with an IC_50_ of 6.3 µg/mL compared to 22.1 µg/mL for free BRB. These findings highlight NLC-B as a promising tumor treatment strategy [26]. Another study developed lyotropic liquid crystalline nanoparticles (LCNs) and demonstrated that these formulations significantly reduced the IC_50_ in MCF7 breast cancer cells compared to free berberine. Cellular uptake assays revealed higher berberine concentrations in Caco-2 cells with NLCs, highlighting their potential as effective carriers for enhancing berberine’s solubility and anticancer activity [75]. Next, we evaluated the safety profiles of NLC, BRB, and NLC-B using L929 (fibroblast-like) and H9c2 (cardiomyocyte-like) cell lines. The non-loaded NLC exhibited minimal cytotoxicity, highlighting its biocompatibility as a carrier system. At the highest experimental concentrations of 1000–2000 µg/mL, cytotoxic effects were observed, but such high concentrations are not supposed to be used in therapy.

As expected, BRB caused dose-dependent cytotoxicity in L929 (fibroblast) and H9c2 (cardiomyocyte) cells. In contrast to free BRB, the encapsulated NLC-B demonstrated less toxicity to the healthy cells. For instance, at 200 µM, NLC-B maintained 62% cell viability in L929 cells versus 29% for free BRB. Similarly, in H9c2 cells, NLC-B showed reduced cardiotoxicity compared to free BRB. This attenuation can be attributed to the controlled release properties of the developed NLC particles, which may prevent acute exposure to high concentrations of free BRB. These findings are crucial for the potential application of NLC-B, as they suggest a safer toxicity profile of the encapsulated NLC-B compared to free BRB. It has been previously reported that NLCs can reach the perinuclear region [76] and also contribute to increased oxidative stress [77]. Up to this moment, there are only a few studies on the genotoxicity of NLCs, and they find only insignificant genotoxic effects [78,79]. In many cases, the presence of genotoxic potential is acceptable for cytotoxic antineoplastic drugs as part of the pharmacological mechanisms. It is known that berberine itself has such effects [80]. Non-active constituents, such as the NLC GRAS lipids, are typically expected to be non-toxic and safe. For this reason, genotoxicity studies were focused on the drug delivery system and not on berberine. The comet assay conducted on L5178 cells revealed that NLC treatment induced DNA fragmentation, indicative of genotoxic effects. However, the extent of damage was significantly lower than that caused by the positive control (hydrogen peroxide). This observation underscores the need for complementary assays to distinguish between genotoxicity and cytotoxicity, as DNA damage could be secondary to cell death. Based on the results from the study, we concluded that the overall safety profile of NLC appears favorable. Nevertheless, the highest concentrations of NLC may induce some degree of oxidative stress, which could explain the observed results from the comet assay [81]. The study performed on isolated rat liver microsomes is related to the metabolic and oxidative interactions of BRB, NLC, and NLC-B. It provides valuable insights into their biochemical properties. The pro-oxidant effects observed at higher concentrations indicate potential risks of lipid peroxidation as a concern for systemic toxicity. However, NLC-B demonstrated lower MDA production compared to free BRB at equimolar concentrations, suggesting a protective effect of the lipid nanocarrier NLC system.

The current work is intended as proof of concept for the synthesis of novel NLCs as drug delivery modalities. It adopts protocols, based on assays, accepted in internationally recognized guidelines and regulatory frameworks as ICH [82], ISO [83] and OECD [84]. It is outside of its scope and it falls short of providing full preclinical characterization of the studied drug delivery system, which would require further tests, including In Vivo studies, set in the current regulatory requirements [85,86]. We have adhered to the Guidance Document on Good In Vitro Method Practices (GIVIMP), which suggests that depending on the specific endpoint, researchers can propose and validate novel batteries of methods, while it is made clear that for research laboratories, it is not realistic to expect full compliance with GIVIMP [87]. A strength of the suggested experimental approach is that it could be applied for quick evaluation and screening of novel drug delivery systems for gastrointestinal tumors. However, the current findings are limited to In Vitro assays and the insights gained could inform future In Vivo research, including pharmacokinetic and bioavailability studies.

## 5. Conclusions

The present research shows the formulation of a nanocarrier system for the improved delivery of the hydrophobic natural compound berberine. The nanostructured lipid carriers (NLCs) were spherical, with high encapsulation efficiency and negative potential, suggesting suitable physicochemical stability. No significant interactions with the excipients were established from a technological point of view. Improved solubility and prolonged dissolution were also evident for the NLC-B. Furthermore, the cytotoxicity studies on two tumor-derived cell lines, HuCC-T1 (human cholangiocarcinoma) and CaCo-2 (colorectal adenocarcinoma) showed an enhanced cytotoxic effect and antiproliferative potential of encapsulated NLC-B. The study findings indicate that NLCs, developed as a drug delivery system, possess a high degree of biocompatibility in non-tumor cells (L929 and H9c2 cells), though their safety is compromised at very high concentrations of 1000 µg/mL and above. Nevertheless, such concentrations are not supposed to be used in future therapeutic applications. These results highlight the value of NLC systems in reducing the adverse effects of cytotoxic drugs while retaining their therapeutic antiproliferative potential.

## Figures and Tables

**Table 1:** Solubility of BRB in different lipids and surfactants.

Type of Excipient	Substance	Solubility
Solid lipid	Precirol^®^ 5 ATO	29.63 mg/g
	Compritol^®^ ATO 888	23.41 mg/g
Liquid lipid	Oleic acid	0.0403 mg/mL
	Mygliol^®^ 812 N	0.4300 mg/mL
	Castor oil	0.3871 mg/mL
Surfactants	Tween 20	0.5342 mg/mL
	Tween 80	0.3160 mg/mL
	Tween 85	0.4231 mg/mL

**Table 2:** Data of the performed dynamic light scattering analysis for the prepared nanocarriers. The values represent mean values of independent batch (1–3) sample measurements ± SD.

Nanoparticles	Z-Average Diameter, nm	PDI	Ζ-Potential, mV
NLC1	168.1 ± 2.3	0.184 ± 0.02	−32.8 ± 1.1
NLC2	167.2 ± 1.8	0.200 ± 0.01	−31.7 ± 2.3
NLC3	169.1 ± 2.1	0.218 ± 0.01	−33.3 ± 1.8
NLC-B1	158.2 ± 1.8	0.305 ± 0.01	−30.7 ± 0.8
NLC-B2	157.7 ± 2.4	0.208 ± 0.02	−29.2 ± 0.5
NLC-B3	159.2 ± 1.7	0.223 ± 0.03	−29.8 ± 1.0

## Data Availability

Original data are contained within the article.

## Supplementary Materials

The following supporting information can be downloaded at: https://www.mdpi.com/article/10.3390/pharmaceutics17030331/s1. Table S1. Thermogravimetric data for Precirol® 5 ATO, berberine, physical mixture (PM), physical mixture with berberine (PMB), empty (NLC) and berberine (NLC-B) loaded nanoparticles. Figure S1. Thermogravimetric curves of Precirol 5 ATO, berberine, physical mixture (PM), physical mixture with berberine (PMB), empty (NLC) and berberine (NLC-B) loaded nanoparticles. Figure S2. Hydrodynamic diameter of the prepared NLC (A–C) and NLC-B (D–F) reported by intensity (A,D), number (B,E) and volume (C,F). Data of three different sample batches.

## References

[1] Vedeld H.M., Goel A., Lind G.E. (2018). Epigenetic Biomarkers in Gastrointestinal Cancers: The Current State and Clinical Perspectives. Semin. Cancer Biol..

[2] Wang K., Feng X., Chai L., Cao S., Qiu F. (2017). The Metabolism of Berberine and Its Contribution to the Pharmacological Effects. Drug Metab. Rev..

[3] Davoodvandi A., Sadeghi S., Alavi S.M.A., Alavi S.S., Jafari A., Khan H., Aschner M., Mirzaei H., Sharifi M., Asemi Z. (2024). The Therapeutic Effects of Berberine for Gastrointestinal Cancers. Asia Pac. J. Clin. Oncol..

[4] Jiang X., Jiang Z., Jiang M., Sun Y. (2022). Berberine as a Potential Agent for the Treatment of Colorectal Cancer. Front. Med..

[5] Hallajzadeh J., Maleki Dana P., Mobini M., Asemi Z., Mansournia M.A., Sharifi M., Yousefi B. (2020). Targeting of Oncogenic Signaling Pathways by Berberine for Treatment of Colorectal Cancer. Med. Oncol. Northwood Lond. Engl..

[6] Niu J., Yuan M., Chen C., Wang L., Tang Z., Fan Y., Liu X., Ma Y.J., Gan Y. (2020). Berberine-Loaded Thiolated Pluronic F127 Polymeric Micelles for Improving Skin Permeation and Retention. Int. J. Nanomed..

[7] Zhaojie M., Ming Z., Shengnan W., Xiaojia B., Hatch G.M., Jingkai G., Li C. (2014). Amorphous Solid Dispersion of Berberine with Absorption Enhancer Demonstrates a Remarkable Hypoglycemic Effect via Improving Its Bioavailability. Int. J. Pharm..

[8] Sun L., Lan J., Li Z., Zeng R., Shen Y., Zhang T., Ding Y. (2024). Transforming Cancer Treatment with Nanotechnology: The Role of Berberine as a Star Natural Compound. Int. J. Nanomed..

[9] Rad S.Z.K., Rameshrad M., Hosseinzadeh H. (2017). Toxicology Effects of Berberis Vulgaris (Barberry) and Its Active Constituent, Berberine: A Review. Iran. J. Basic Med. Sci..

[10] Huang Z., Hua S., Yang Y., Fang J. (2008). Development and Evaluation of Lipid Nanoparticles for Camptothecin Delivery: A Comparison of Solid Lipid Nanoparticles, Nanostructured Lipid Carriers, and Lipid Emulsion. Acta Pharmacol. Sin..

[11] Kohli K., Mujtaba A., Malik R., Amin S., Alam M.S., Ali A., Barkat M.A., Ansari M.J. (2021). Development of Natural Polysaccharide–Based Nanoparticles of Berberine to Enhance Oral Bioavailability: Formulation, Optimization, Ex Vivo, and In Vivo Assessment. Polymers.

[12] Kutbi H.I., Asfour H.Z., Kammoun A.K., Sirwi A., Cavalu S., Gad H.A. (2021). Optimization of Hyaluronate-Based Liposomes to Augment the Oral Delivery and the Bioavailability of Berberine. Materials.

[13] Mirhadi E., Rezaee M., Malaekeh-Nikouei B. (2018). Nano Strategies for Berberine Delivery, a Natural Alkaloid of Berberis. Biomed. Pharmacother..

[14] Ryu S., Jin M., Lee H.-K., Wang M.-H., Baek J.-S., Cho C.-W. (2022). Effects of Lipid Nanoparticles on Physicochemical Properties, Cellular Uptake, and Lymphatic Uptake of 6-Methoxflavone. J. Pharm. Investig..

[15] Blanco-Llamero C., Fonseca J., Durazzo A., Lucarini M., Santini A., Señoráns F.J., Souto E.B. (2022). Nutraceuticals and Food-Grade Lipid Nanoparticles: From Natural Sources to a Circular Bioeconomy Approach. Foods.

[16] Abdel-Mageed H.M., Abd El Aziz A.E., Mohamed S.A., AbuelEzz N.Z. (2022). The Tiny Big World of Solid Lipid Nanoparticles and Nanostructured Lipid Carriers: An Updated Review. J. Microencapsul..

[17] Jadon R.S., Jadon P.S., Bhadauria V., Sharma V., Bharadwaj S., Sharma M., Gajbhiye K.R., Gajbhiye V., Gajbhiye V., Gajbhiye K.R., Hong S. (2022). Chapter 10—Solid–Lipid Nanoparticles Based Vehicles for Stimuli Inspired Delivery of Bioactives. Stimuli-Responsive Nanocarriers.

[18] Wilhelm S., Tavares A.J., Dai Q., Ohta S., Audet J., Dvorak H.F., Chan W.C.W. (2016). Analysis of Nanoparticle Delivery to Tumours. Nat. Rev. Mater..

[19] Nguyen L.N.M., Ngo W., Lin Z., Sindhwani S., MacMillan P., Mladjenovic S., Chan W. (2024). The Mechanisms of Nanoparticle Delivery to Solid Tumours. Nat. Rev. Bioeng..

[20] Asha T., Seema K., Sanjeevani D., Lata K., Sohan C. (2021). Bioavailability of Berberine: Challenges and Solutions. İstanbul J. Pharm..

[21] Nagpal S., Png Yi Jie J., Malinovskaya J., Kovshova T., Jain P., Naik S., Khopade A., Bhowmick S., Shahi P., Chakra A. (2024). A Design-Conversed Strategy Establishes the Performance Safe Space for Doxorubicin Nanosimilars. ACS Nano.

[22] Madkhali O.A. (2022). Perspectives and Prospective on Solid Lipid Nanoparticles as Drug Delivery Systems. Molecules.

[23] Mao M., Liu S., Zhou Y., Wang G., Deng J., Tian L. (2020). Nanostructured Lipid Carrier Delivering Chlorins E6 as in Situ Dendritic Cell Vaccine for Immunotherapy of Gastric Cancer. J. Mater. Res..

[24] Chauhan I., Yasir M., Verma M., Singh A.P. (2020). Nanostructured Lipid Carriers: A Groundbreaking Approach for Transdermal Drug Delivery. Adv. Pharm. Bull..

[25] Nguyen T.-T.-L., Duong V.-A. (2022). Solid Lipid Nanoparticles. Encyclopedia.

[26] Wang Z., Wu J., Chen T., Zhou Q., Wang Y. In Vitro and In Vivo Antitumor Efficacy of Berberine-Nanostructured Lipid Carriers against H22 Tumor. Proceedings of the Biophotonics and Immune Responses X.

[27] Shirodkar R.K., Kumar L., Mutalik S., Lewis S. (2019). Solid Lipid Nanoparticles and Nanostructured Lipid Carriers: Emerging Lipid Based Drug Delivery Systems. Pharm. Chem. J..

[28] Tenchov R., Bird R., Curtze A.E., Zhou Q. (2021). Lipid Nanoparticles─From Liposomes to mRNA Vaccine Delivery, a Landscape of Research Diversity and Advancement. ACS Nano.

[29] Teixeira M.C., Carbone C., Souto E.B. (2017). Beyond Liposomes: Recent Advances on Lipid Based Nanostructures for Poorly Soluble/Poorly Permeable Drug Delivery. Prog. Lipid Res..

[30] Talegaonkar S., Bhattacharyya A. (2019). Potential of Lipid Nanoparticles (SLNs and NLCs) in Enhancing Oral Bioavailability of Drugs with Poor Intestinal Permeability. AAPS PharmSciTech.

[31] Nagpal S., Palaniappan T., Wang J.-W., Wacker M.G. (2024). Revisiting Nanomedicine Design Strategies for Follow-on Products: A Model-Informed Approach to Optimize Performance. J. Control. Release.

[32] Doktorovová S., Kovačević A.B., Garcia M.L., Souto E.B. (2016). Preclinical Safety of Solid Lipid Nanoparticles and Nanostructured Lipid Carriers: Current Evidence from *In Vitro* and *In Vivo* Evaluation. Eur. J. Pharm. Biopharm..

[33] Ahmed T., Gilani A.-U.-H., Abdollahi M., Daglia M., Nabavi S.F., Nabavi S.M. (2015). Berberine and Neurodegeneration: A Review of Literature. Pharmacol. Rep. PR.

[34] Raju M., Kunde S.S., Auti S.T., Kulkarni Y.A., Wairkar S. (2021). Berberine Loaded Nanostructured Lipid Carrier for Alzheimer’s Disease: Design, Statistical Optimization and Enhanced In Vivo Performance. Life Sci..

[35] Sakellari G.I., Zafeiri I., Batchelor H., Spyropoulos F. (2021). Formulation Design, Production and Characterisation of Solid Lipid Nanoparticles (SLN) and Nanostructured Lipid Carriers (NLC) for the Encapsulation of a Model Hydrophobic Active. Food Hydrocoll. Health.

[36] Deng J., Wu Z., Zhao Z., Wu C., Yuan M., Su Z., Wang Y., Wang Z. (2020). Berberine-Loaded Nanostructured Lipid Carriers Enhance the Treatment of Ulcerative Colitis. Int. J. Nanomed..

[37] Gu L., Sun R., Wang W., Xia Q. (2022). Nanostructured Lipid Carriers for the Encapsulation of Phloretin: Preparation and In Vitro Characterization Studies. Chem. Phys. Lipids.

[38] Gomaa E., Fathi H.A., Eissa N.G., Elsabahy M. (2022). Methods for Preparation of Nanostructured Lipid Carriers. Methods.

[39] Shen J., Burgess D.J. (2013). In Vitro Dissolution Testing Strategies for Nanoparticulate Drug Delivery Systems: Recent Developments and Challenges. Drug Deliv. Transl. Res..

[40] Fathi H.A., Allam A., Elsabahy M., Fetih G., El-Badry M. (2018). Nanostructured Lipid Carriers for Improved Oral Delivery and Prolonged Antihyperlipidemic Effect of Simvastatin. Colloids Surf. B Biointerfaces.

[41] Lohan S., Sharma T., Saini S., Singh A., Kumar A., Raza K., Kaur J., Singh B. (2021). Galactosylated Nanoconstructs of Berberine with Enhanced Biopharmaceutical and Cognitive Potential: A Preclinical Evidence in Alzheimer ‘s Disease. J. Drug Deliv. Sci. Technol..

[42] D’Souza S. (2014). A Review of In Vitro Drug Release Test Methods for Nano-Sized Dosage Forms. Adv. Pharm..

[43] Mosmann T. (1983). Rapid Colorimetric Assay for Cellular Growth and Survival: Application to Proliferation and Cytotoxicity Assays. J. Immunol. Methods.

[44] O’Brien J., Wilson I., Orton T., Pognan F. (2000). Investigation of the Alamar Blue (Resazurin) Fluorescent Dye for the Assessment of Mammalian Cell Cytotoxicity. Eur. J. Biochem..

[45] Clementi E., Garajova Z., Markkanen E. (2021). Measuring DNA Damage Using the Alkaline Comet Assay in Cultured Cells. Bio-Protocol.

[46] Schindelin J., Arganda-Carreras I., Frise E., Kaynig V., Longair M., Pietzsch T., Preibisch S., Rueden C., Saalfeld S., Schmid B. (2012). Fiji: An Open-Source Platform for Biological-Image Analysis. Nat. Methods.

[47] Gyori B.M., Venkatachalam G., Thiagarajan P.S., Hsu D., Clement M.-V. (2014). OpenComet: An Automated Tool for Comet Assay Image Analysis. Redox Biol..

[48] Simeonova R.L., Vitcheva V.B., Kondeva-Burdina M.S., Krasteva I.N., Nikolov S.D., Mitcheva M.K. (2010). Effect of Purified Saponin Mixture from *Astragalus Corniculatus* on Enzyme- and Non-Enzyme-Induced Lipid Peroxidation in Liver Microsomes from Spontaneously Hypertensive Rats and Normotensive Rats. Phytomedicine.

[49] Lowry O.H., Rosebrough N.J., Farr A.L., Randall R.J. (1951). Protein Measurement with the Folin Phenol Reagent. J. Biol. Chem..

[50] Kim H.J., Chun Y.J., Park J.D., Kim S.I., Roh J.K., Jeong T.C. (1997). Protection of Rat Liver Microsomes against Carbon Tetrachloride-Induced Lipid Peroxidation by Red Ginseng Saponin through Cytochrome P450 Inhibition. Planta Med..

[51] Younis F.A., Saleh S.R., El-Rahman S.S.A., Newairy A.-S.A., El-Demellawy M.A., Ghareeb D.A. (2022). Preparation, Physicochemical Characterization, and Bioactivity Evaluation of Berberine-Entrapped Albumin Nanoparticles. Sci. Rep..

[52] Bashmakova N., Kutovyy S., Zhurakivsky R., Hovorun D., Yashchuk V. (2011). Koливальний спектр oрганічнoї спoлуки берберину та йoгo інтерпретація квантoвo-механічним метoдoм функціoнала густини. Ukr. J. Phys..

[53] Vitorino C., Silva S., Gouveia F., Bicker J., Falcão A., Fortuna A. (2020). QbD-Driven Development of Intranasal Lipid Nanoparticles for Depression Treatment. Eur. J. Pharm. Biopharm..

[54] Teixeira M.I., Lopes C.M., Gonçalves H., Catita J., Silva A.M., Rodrigues F., Amaral M.H., Costa P.C. (2022). Formulation, Characterization, and Cytotoxicity Evaluation of Lactoferrin Functionalized Lipid Nanoparticles for Riluzole Delivery to the Brain. Pharmaceutics.

[55] Domingues C., Santos A., Alvarez-Lorenzo C., Concheiro A., Jarak I., Veiga F., Barbosa I., Dourado M., Figueiras A. (2022). Where Is Nano Today and Where Is It Headed? A Review of Nanomedicine and the Dilemma of Nanotoxicology. ACS Nano.

[56] Chellat F., Tabrizian M., Dumitriu S., Chornet E., Magny P., Rivard C.H., Yahia L. (2000). In Vitro and In Vivo Biocompatibility of Chitosan-Xanthan Polyionic Complex. J. Biomed. Mater. Res..

[57] Sangweni N.F., Moremane M., Riedel S., van Vuuren D., Huisamen B., Mabasa L., Barry R., Johnson R. (2020). The Prophylactic Effect of Pinocembrin Against Doxorubicin-Induced Cardiotoxicity in an In Vitro H9c2 Cell Model. Front. Pharmacol..

[58] Li A.P. (2005). Preclinical In Vitro Screening Assays for Drug-like Properties. Drug Discov. Today Technol..

[59] Pelkonen O., Kaltiala E.H., Larmi T.K., Kärki N.T. (1974). Cytochrome P-450-Linked Monooxygenase System and Drug-Induced Spectral Interactions in Human Liver Microsomes. Chem. Biol. Interact..

[60] Eichelbaum M., Burk O. (2001). CYP3A Genetics in Drug Metabolism. Nat. Med..

[61] Arnold M., Abnet C.C., Neale R.E., Vignat J., Giovannucci E.L., McGlynn K.A., Bray F. (2020). Global Burden of 5 Major Types of Gastrointestinal Cancer. Gastroenterology.

[62] Kaur R., Bhardwaj A., Gupta S. (2023). Cancer Treatment Therapies: Traditional to Modern Approaches to Combat Cancers. Mol. Biol. Rep..

[63] Behl T., Singh S., Sharma N., Zahoor I., Albarrati A., Albratty M., Meraya A.M., Najmi A., Bungau S. (2022). Expatiating the Pharmacological and Nanotechnological Aspects of the Alkaloidal Drug Berberine: Current and Future Trends. Molecules.

[64] Wu J. (2021). The Enhanced Permeability and Retention (EPR) Effect: The Significance of the Concept and Methods to Enhance Its Application. J. Pers. Med..

[65] Ke Z., Zhu Z., Xu Z., Fang C., Hu S. (2015). Formulation Design and In Vitro Evaluation of Berberine- Loaded Self-Nanoemulsifying Drug Delivery System. Trop. J. Pharm. Res..

[66] Onugwu A.L., Nwagwu C.S., Onugwu O.S., Echezona A.C., Agbo C.P., Ihim S.A., Emeh P., Nnamani P.O., Attama A.A., Khutoryanskiy V.V. (2023). Nanotechnology Based Drug Delivery Systems for the Treatment of Anterior Segment Eye Diseases. J. Control. Release.

[67] Kovacevic A., Savic S., Vuleta G., Müller R.H., Keck C.M. (2011). Polyhydroxy Surfactants for the Formulation of Lipid Nanoparticles (SLN and NLC): Effects on Size, Physical Stability and Particle Matrix Structure. Int. J. Pharm..

[68] Abolhasanzadeh N., Dehghan G., Abbaspour-Ravasjani S. (2024). Enhancement of the Stability and Cytotoxicity of Berberine by Liposomal Nanocarriers for Gastric Cancer Treatment and Its Application in Gummy Candy. Front. Gastroenterol..

[69] Gendy A.M., Elnagar M.R., Allam M.M., Mousa M.R., Khodir A.E., El-Haddad A.E., Elnahas O.S., Fayez S.M., El-Mancy S.S. (2022). Berberine-Loaded Nanostructured Lipid Carriers Mitigate Warm Hepatic Ischemia/Reperfusion-Induced Lesion through Modulation of HMGB1/TLR4/NF-κB Signaling and Autophagy. Biomed. Pharmacother. Biomed. Pharmacother..

[70] How C.W., Rasedee A., Manickam S., Rosli R. (2013). Tamoxifen-Loaded Nanostructured Lipid Carrier as a Drug Delivery System: Characterization, Stability Assessment and Cytotoxicity. Colloids Surf. B Biointerfaces.

[71] Laracuente M.-L., Yu M.H., McHugh K.J. (2020). Zero-Order Drug Delivery: State of the Art and Future Prospects. J. Control. Release.

[72] Committee E.S. (2011). Scientific Opinion on Genotoxicity Testing Strategies Applicable to Food and Feed Safety Assessment. EFSA J..

[73] Hesari A., Ghasemi F., Cicero A.F.G., Mohajeri M., Rezaei O., Hayat S.M.G., Sahebkar A. (2018). Berberine: A Potential Adjunct for the Treatment of Gastrointestinal Cancers?. J. Cell. Biochem..

[74] He W., Wang B., Zhuang Y., Shao D., Sun K., Chen J. (2012). Berberine Inhibits Growth and Induces G1 Arrest and Apoptosis in Human Cholangiocarcinoma QBC939 Cells. J. Pharmacol. Sci..

[75] Loo Y.S., Madheswaran T., Rajendran R., Bose R.J.c. (2020). Encapsulation of Berberine into Liquid Crystalline Nanoparticles to Enhance Its Solubility and Anticancer Activity in MCF7 Human Breast Cancer Cells. J. Drug Deliv. Sci. Technol..

[76] Tan A., Hong L., Du J.D., Boyd B.J. (2019). Self-Assembled Nanostructured Lipid Systems: Is There a Link between Structure and Cytotoxicity?. Adv. Sci. Weinh. Baden-Wurtt. Ger..

[77] Silva A.H., Filippin-Monteiro F.B., Mattei B., Zanetti-Ramos B.G., Creczynski-Pasa T.B. (2012). In Vitro Biocompatibility of Solid Lipid Nanoparticles. Sci. Total Environ..

[78] Doktorovova S., Silva A.M., Gaivão I., Souto E.B., Teixeira J.P., Martins-Lopes P. (2014). Comet Assay Reveals No Genotoxicity Risk of Cationic Solid Lipid Nanoparticles. J. Appl. Toxicol. JAT.

[79] Doktorovova S., Souto E.B., Silva A.M. (2014). Nanotoxicology Applied to Solid Lipid Nanoparticles and Nanostructured Lipid Carriers—A Systematic Review of In Vitro Data. Eur. J. Pharm. Biopharm. Off. J. Arbeitsgemeinschaft Pharm. Verfahrenstechnik EV.

[80] Hou D., Xu G., Zhang C., Li B., Qin J., Hao X., Liu Q., Zhang X., Liu J., Wei J. (2017). Berberine Induces Oxidative DNA Damage and Impairs Homologous Recombination Repair in Ovarian Cancer Cells to Confer Increased Sensitivity to PARP Inhibition. Cell Death Dis..

[81] Cho E., Allemang A., Audebert M., Chauhan V., Dertinger S., Hendriks G., Luijten M., Marchetti F., Minocherhomji S., Pfuhler S. (2022). AOP Report: Development of an Adverse Outcome Pathway for Oxidative DNA Damage Leading to Mutations and Chromosomal Aberrations. Environ. Mol. Mutagen..

[82] ICH S2 (R1) Genotoxicity Testing and Data Interpretation for Pharmaceuticals Intended for Human Use—Scientific Guideline|European Medicines Agency (EMA). https://www.ema.europa.eu/en/ich-s2-r1-genotoxicity-testing-data-interpretation-pharmaceuticals-intended-human-use-scientific-guideline.

[83] ISO 10993-5:2009. https://www.iso.org/standard/36406.html.

[84] Guidance Document on Using Cytotoxicity Tests to Estimate Starting Doses for Acute Oral Systematic Toxicity Tests|OECD. https://www.oecd.org/en/publications/guidance-document-on-using-cytotoxicity-tests-to-estimate-starting-doses-for-acute-oral-systematic-toxicity-tests_d77a7e39-en.html.

[85] ICH M3 (R2) Non-Clinical Safety Studies for the Conduct of Human Clinical Trials for Pharmaceuticals—Scientific Guideline|European Medicines Agency (EMA). https://www.ema.europa.eu/en/ich-m3-r2-non-clinical-safety-studies-conduct-human-clinical-trials-pharmaceuticals-scientific-guideline.

[86] ICH S9 Non-Clinical Evaluation for Anticancer Pharmaceuticals—Scientific Guideline|European Medicines Agency (EMA). https://www.ema.europa.eu/en/ich-s9-non-clinical-evaluation-anticancer-pharmaceuticals-scientific-guideline.

[87] Guidance Document on Good In Vitro Method Practices (GIVIMP)|OECD. https://www.oecd.org/en/publications/guidance-document-on-good-in-vitro-method-practices-givimp_9789264304796-en.html.

