# Supplementary material for: In Vitro Evaluation of the Safety and Antineoplastic Effects in Gastrointestinal Tumors of Nanostructured Lipid Carriers Loaded with Berberine
Source: Pharmaceutics. 2025 Mar 4;17(3):331. doi: 10.3390/pharmaceutics17030331 (PMC11945150; doi:10.3390/pharmaceutics17030331)
Supplement: Supplementary file 1 [file pharmaceutics-17-00331-s001.zip › pharmaceutics-3424009-supplementary.pdf]

Supplementary materials

In Vitro Evaluation of the Safety and Antineoplastic Effects in Gastrointestinal Tumors of Nanostructured Lipid Carriers Loaded with Berberine

Denitsa Stefanova [1], Yordan Yordanov [1], Radostina Bogdanova [1], Christina Voycheva [2],*, Borislav Tzankov [2], Teodora Popova [2], Magdalena Kondeva-Budina [1], Virginia Tzankova [1], Natalia Toncheva-Moncheva [3], Diana Tzankova [4] and Marta Slavkova [2]

[1] Department of Pharmacology, Pharmacotherapy and Toxicology, Faculty of Pharmacy, Medical University of Sofia, 1000 Sofia, Bulgaria; denitsa.stefanova@pharmfac.mu-sofia.bg (D.S.); yyordanov@pharmfac.mu-sofia.bg (Y.Y.); radostina.bogdanovaa@gmail.com (R.B.); mkondeva@pharmfac.mu-sofia.bg (M.K.-B.); vtzankova@pharmfac.mu-sofia.bg (V.T.)

[2] Department of Pharmaceutical Technology and Biopharmaceutics, Faculty of Pharmacy, Medical University of Sofia, 1000 Sofia, Bulgaria; btzankov@pharmfac.mu-sofia.bg (B.T.); tpopova@pharmfac.mu-sofia.bg (T.P.); mslavkova@pharmfac.mu-sofia.bg (M.S.)

[3] Institute of Polymers, Bulgarian Academy of Sciences, bl.103 Akad. G. Bonchev Str., 1113 Sofia, Bulgaria; ntoncheva@polymer.bas.bg

[4] Department of Pharmaceutical Chemistry, Faculty of Pharmacy, Medical University—Sofia, 1000 Sofia, Bulgaria; d.tsankova@pharmfac.mu-sofia.bg

* Correspondence: hvoycheva@pharmfac.mu-sofia.bg; Tel.: +359-2-9236571

Thermogravimetric analyses (TGA)

TGA were performed on a Perkin Elmer TGA 4000 (Waltham, MA, USA). Measurements were run at a heating rate of 10 °C.min^{-1} under a nitrogen flow rate of 20 mL.min^{-1}, to avoid any thermo-oxidative degradation. Instrument control, data acquisition, and data processing were performed by Pyris software (v.11.0.0.0449). The ±1 °C accuracy on the degradation temperatures determined from the derivatives of the weight losses versus temperature curves was established.

The Precirol® 5 ATO (Prec), shows a one-step degradation curve in contrast to a two-step process when physical mixture (PM), physical mixture with berberine (PMB), empty (NLC) and berberine-loaded (NLC-B) nanoparticles were investigated. The TGA of free berberine (BRB), reveal multi-step degradation and presence around 13% moisture in the sample. Both the initial degradation temperature ($T_{d5\%)}$ and the temperature at the maximum weight loss (T_{dmax}) are presented in Table 1.

Table S1. Thermogravimetric data for Precirol® 5 ATO, berberine, physical mixture (PM), physical mixture with berberine (PMB), empty (NLC) and berberine (NLC-B) loaded nanoparticles.

№	Sample code	$T_{d5\%}$ (°C)^a	T_{dmax} (°C)^b
1.	Prec	200	400
2.	BRB	170	-
3.	PM	238	405
4.	PMB	225	405

**5.**	**NLC**	**238**	**420**
**6.**	**NLC-B**	**195**	**420**

[a] $T_{d5\%}$ – Initial degradation temperature that corresponds to weight loss of 5%.

[b] T_{dmax}, V_{dmax} – Maximum degradation temperature and maximum degradation rate that correspond to weight loss of 50%.

[c] $T_{d15\%}$ – Initial degradation temperature that corresponds to weight loss of 15%. The calculated 13% weight loss up to 100 °C is due to moisture content in the sample.

The presented data in Table A1. reveal that all samples are thermally stable up to 170 °C for BRB and 195÷200 °C for other investigated samples. On that base the melting and crystallization behaviour were studied by DSC in the -70 to 170 or 195÷200 °C temperature range.

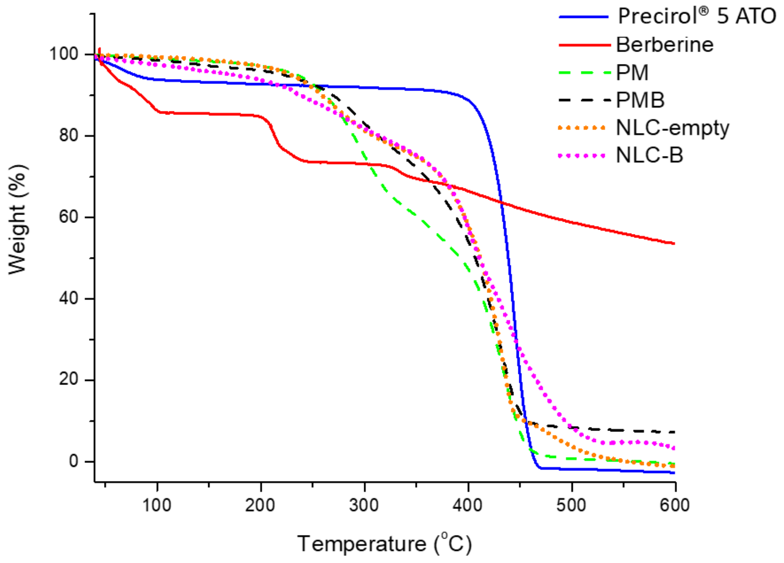

Figure S1. Thermogravimetric curves of Precirol 5 ATO, berberine, physical mixture (PM), physical mixture with berberine (PMB), empty (NLC) and berberine (NLC-B) loaded nanoparticles.

Filename: C:\Program Files\PerkinElmer\P...\Prec1.t6d
Operator ID: RR
Sample ID: Prec
Sample Weight: 4.230 mg
Comment:

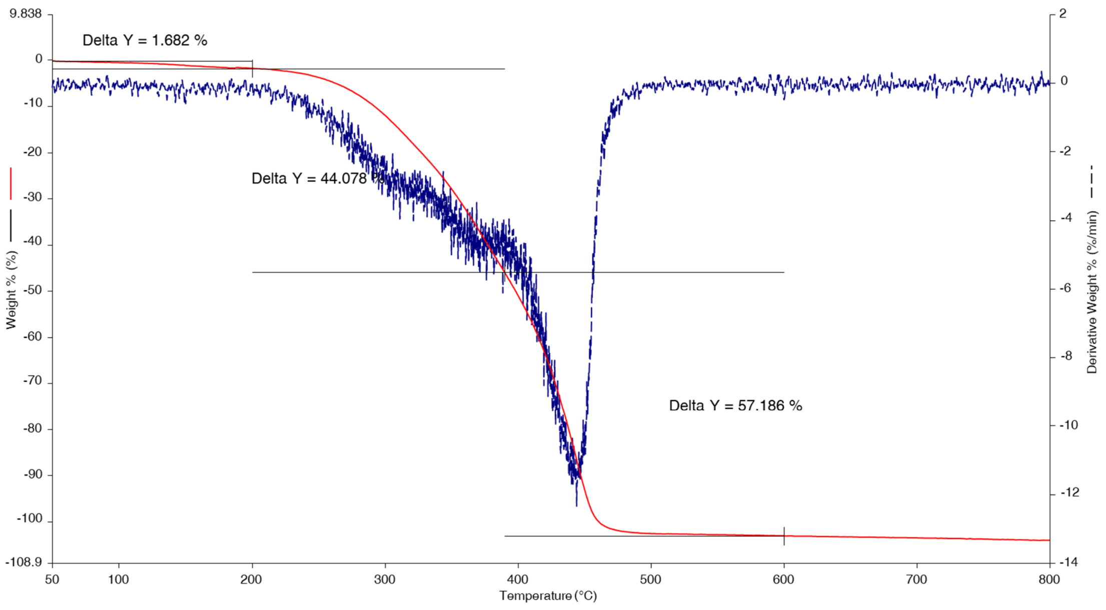

7/22/2024 4:23:24 PM

1) Heat from 40.00°C to 800.00°C at 10.00°C/min 2) Hold for 2.0 min at 800.00°C

Filename: C:\Program Files...\alumel@240716141547.t6d
Operator ID: RR
Sample ID: Brb5
Sample Weight: 2.940 mg
Comment:

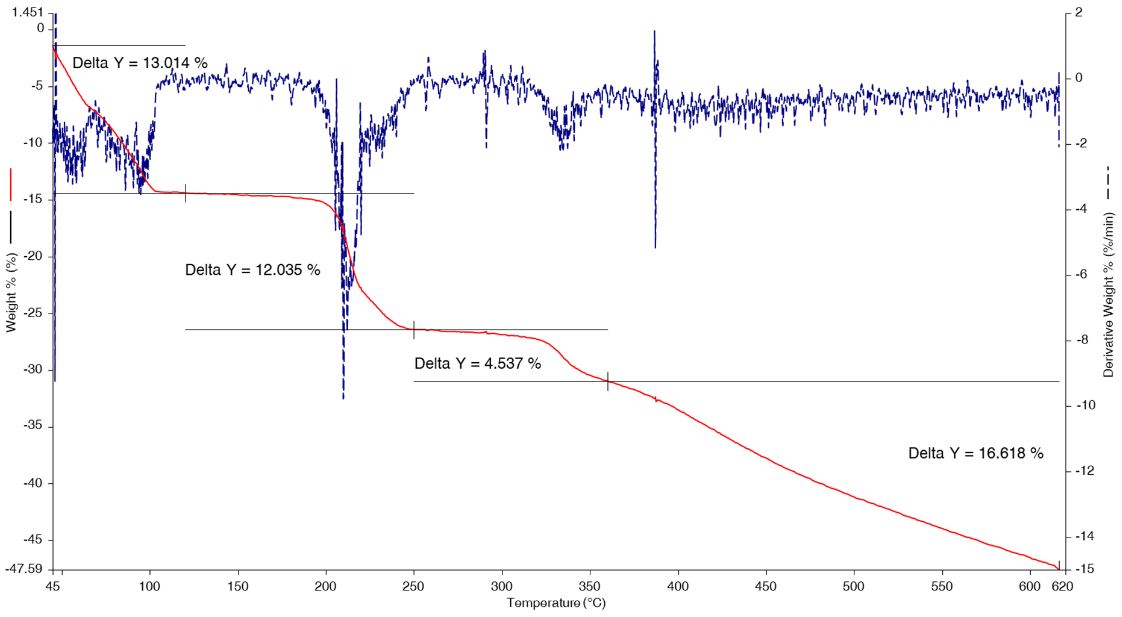

7/22/2024 4:21:24 PM

1) Heat from 40.00°C to 600.00°C at 10.00°C/min 2) Hold for 1.0 min at 600.00°C

Filename: C:\Program Files\PerkinElmer\Pyr...\PNE.t6d
Operator ID: RR
Sample ID: PNE Physical Mixture – nanoparticles components
Sample Weight: 11.750 mg
Comment:

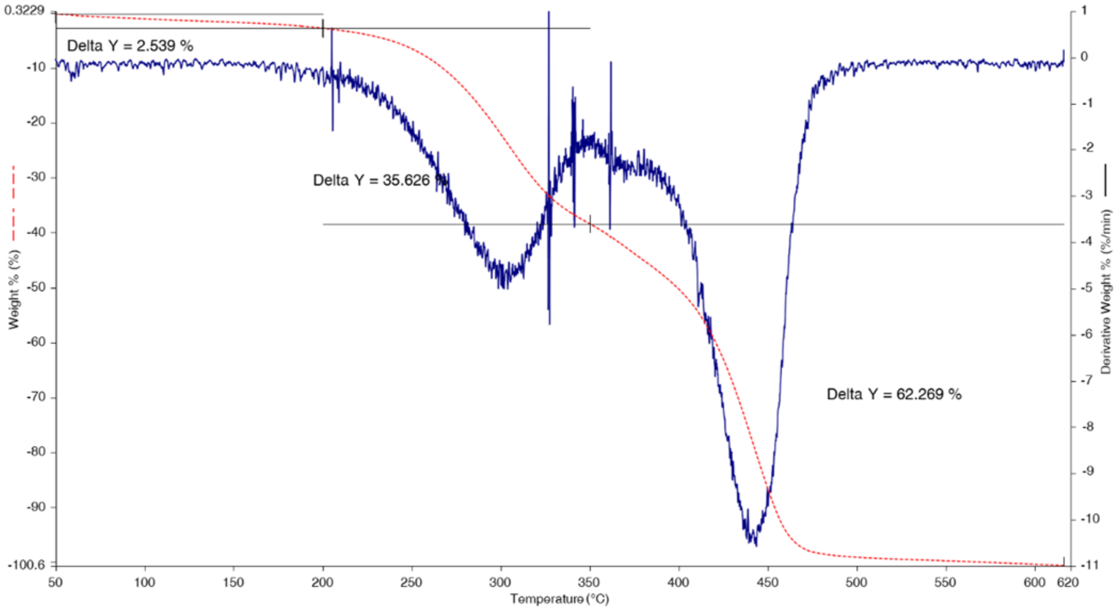

7/22/2024 4:27:41 PM

1) Heat from 40.00°C to 600.00°C at 10.00°C/min 2) Hold for 1.0 min at 600.00°C

Filename: C:\Program Files\PerkinElmer\P...\PNB_1.t6d
Operator ID: RR
Sample ID: PNB_1 Physical mixture with Berberin
Sample Weight: 11.450 mg
Comment:

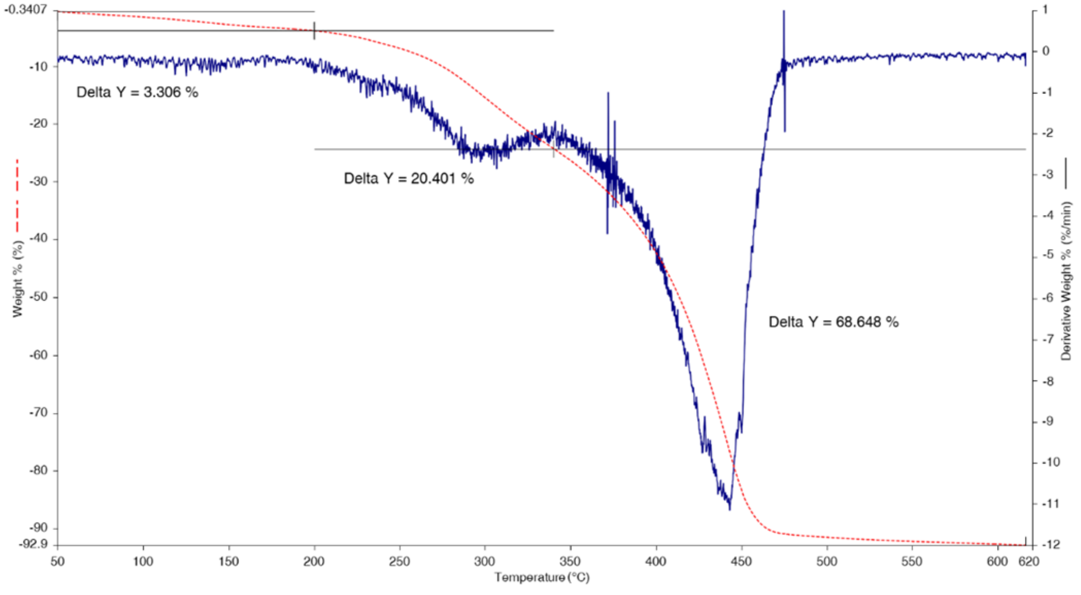

7/22/2024 4:26:03 PM

1) Heat from 40.00°C to 600.00°C at 10.00°C/min 2) Hold for 1.0 min at 600.00°C

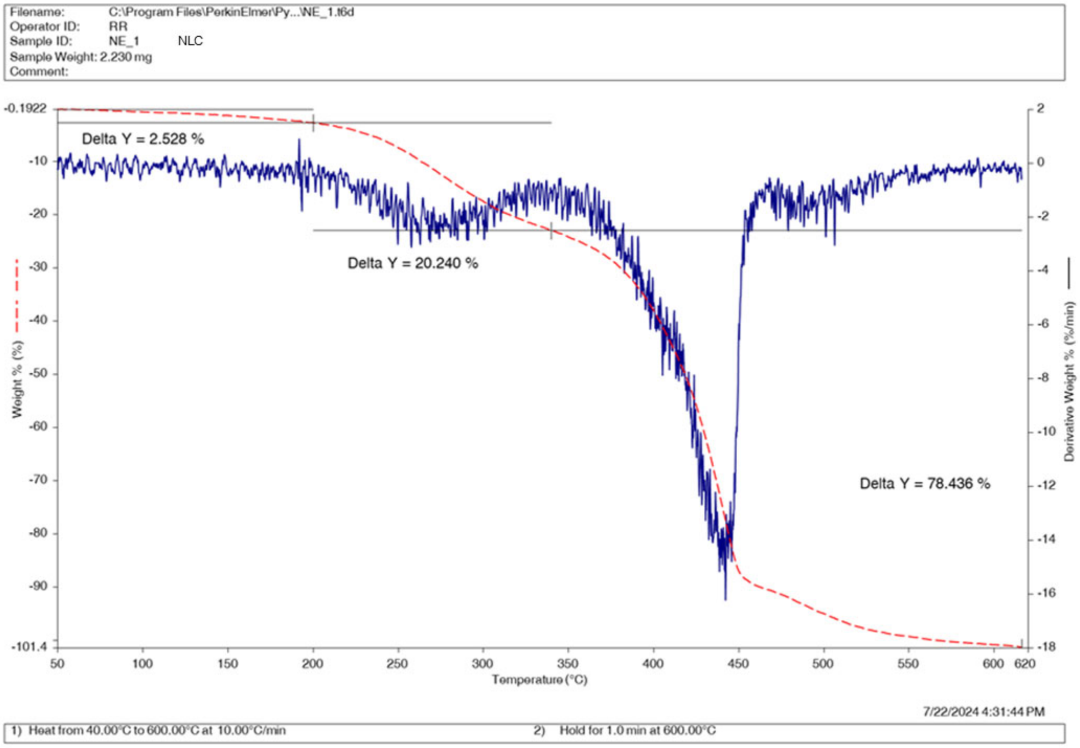
Filename: C:\Program Files\PerkinElmer\Py...\NE_1.t6d
Operator ID: RR
Sample ID: NE_1 NLC
Sample Weight: 2.230 mg
Comment:
Delta Y = 2.528 %
Delta Y = 20.240 %
Delta Y = 78.436 %
Weight % (%)
Derivative Weight % (%/min)
Temperature (°C)
-0.1922
-101.4
7/22/2024 4:31:44 PM
1) Heat from 40.00°C to 600.00°C at 10.00°C/min
2) Hold for 1.0 min at 600.00°C

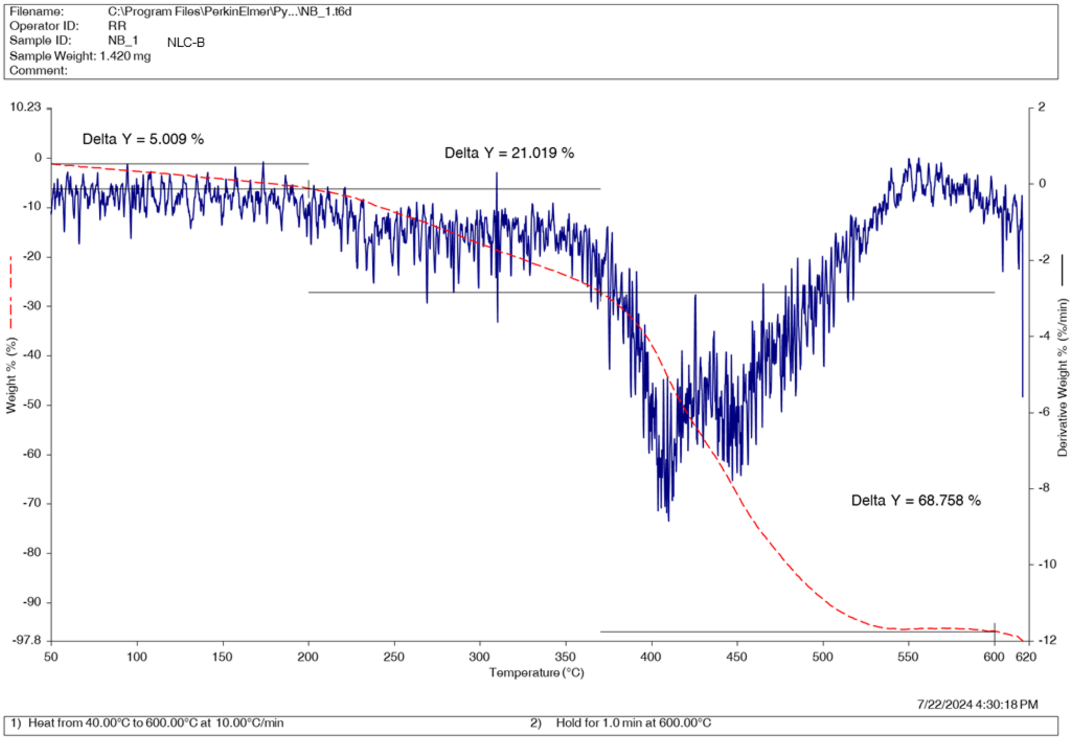
Filename: C:\Program Files\PerkinElmer\Py...\NB_1.t6d
Operator ID: RR
Sample ID: NB_1 NLC-B
Sample Weight: 1.420 mg
Comment:
Delta Y = 5.009 %
Delta Y = 21.019 %
Delta Y = 68.758 %
Weight % (%)
Derivative Weight % (%/min)
Temperature (°C)
10.23
-97.8
7/22/2024 4:30:18 PM
1) Heat from 40.00°C to 600.00°C at 10.00°C/min
2) Hold for 1.0 min at 600.00°C

Figure S2. Hydrodynamic diameter of the prepared NLC (A, B and C) and NLC-B (D, E and F) reported by intensity (A and D), number (B and E) and volume (C and F). Data of three different sample batches.

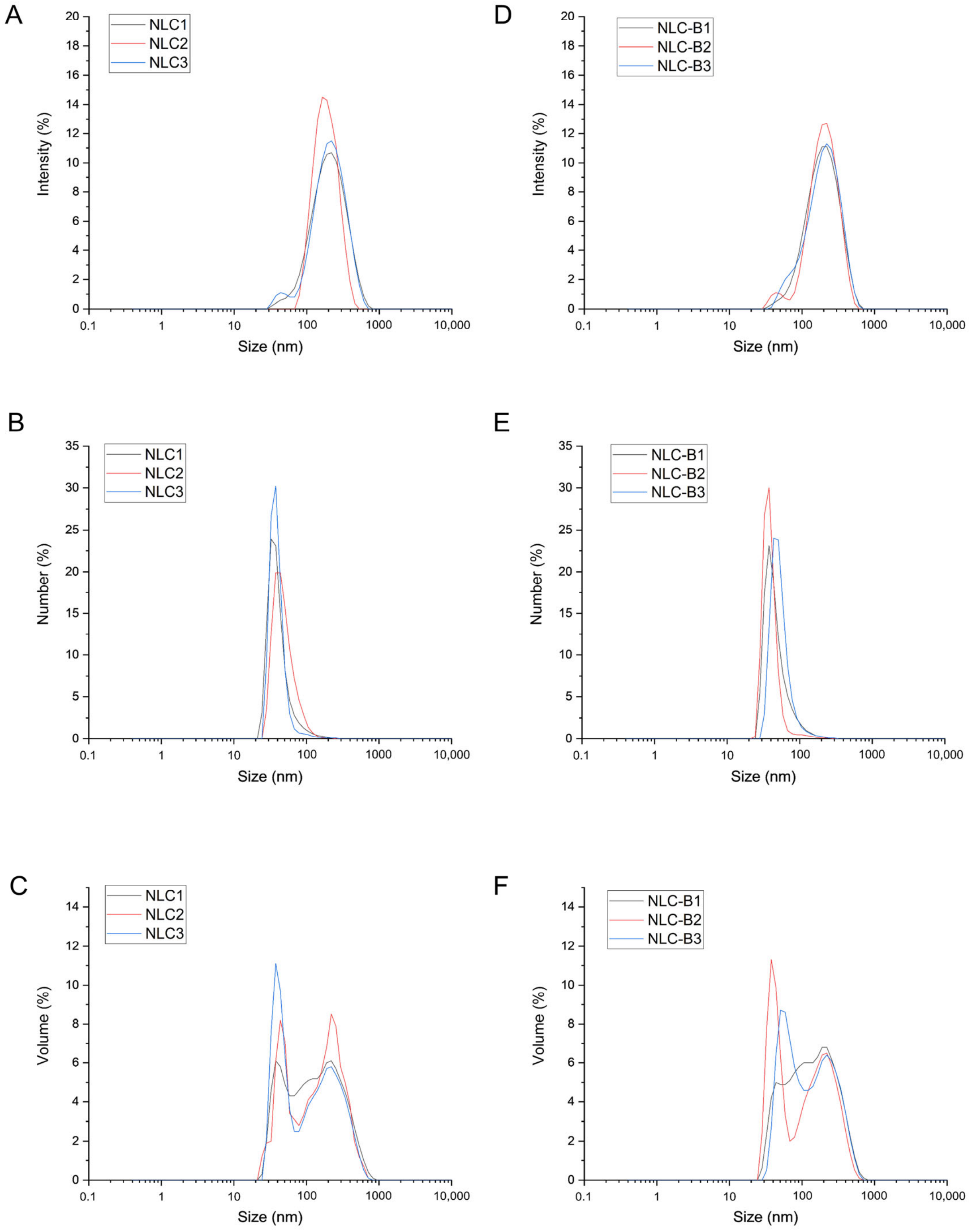

The FTIR spectra of the individual components, plain NLC, and berberine-loaded NLC (NLC-B) are added to the supplementary information. These spectra include:

1. Pure Berberine Hydrochloride (BRB):
    - 3057 cm^{-1}: Quaternary ammonium group.
    - 2910 cm^{-1}: C-H stretching.
    - 2855 cm^{-1}: Methoxyl ($-OCH_3$) group.
    - 1627 cm^{-1}: C-N band.
    - 1601 cm^{-1}: Quaternary iminium ion ($C=N^+$).
    - 1507 cm^{-1}: Aromatic C-C bond vibration.
    - 1110 cm^{-1}: Ring deformation and CH in-plane bending.
    - 1039 cm^{-1}: C-H vibrations in the aromatic ring.
2. Lipid Components:
    - Precirol® 5 ATO:
        - 1730 cm^{-1}: C=O stretching.
        - 2914 cm^{-1} and 2850 cm^{-1}: C-H stretching.
    - Mygliol® 812N:
        - 1738 cm^{-1}: C=O stretching.
        - 2846 cm^{-1} and 2926 cm^{-1}: C-H stretching.
3. NLC and NLC-B:
    - NLC: Combined peaks from Precirol® 5 ATO and Mygliol® 812N without evidence of interactions between lipids.
    - NLC-B: Disappearance of berberine-specific peaks (e.g., aromatic C-C and C-H vibrations, and the quaternary iminium ion), indicating successful encapsulation.

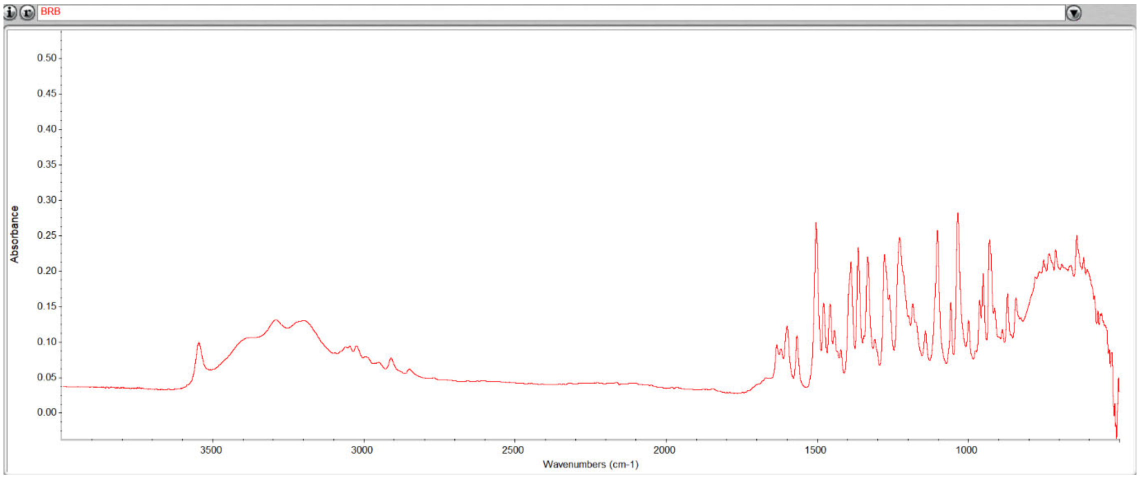

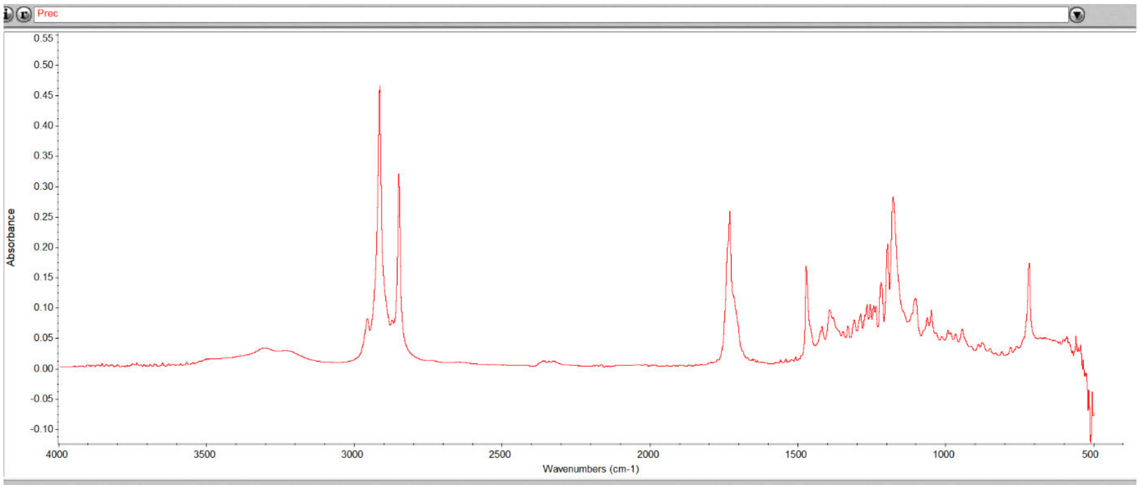
Prec
0.55
0.50
0.45
0.40
0.35
0.30
0.25
0.20
0.15
0.10
0.05
0.00
-0.05
-0.10
Absorbance
4000
3500
3000
2500
2000
1500
1000
500
Wavenumbers (cm-1)

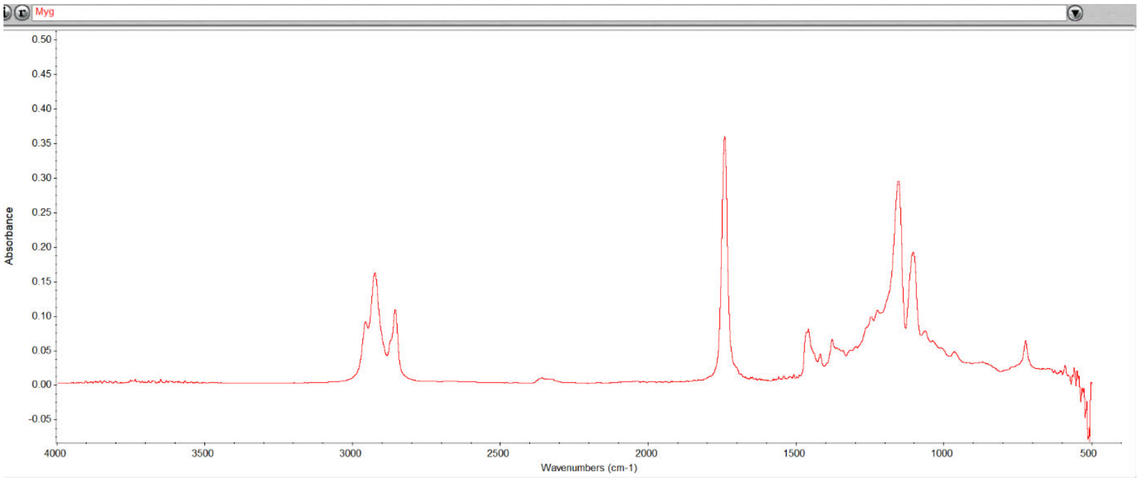
Myg
0.50
0.45
0.40
0.35
0.30
0.25
0.20
0.15
0.10
0.05
0.00
-0.05
Absorbance
4000
3500
3000
2500
2000
1500
1000
500
Wavenumbers (cm-1)

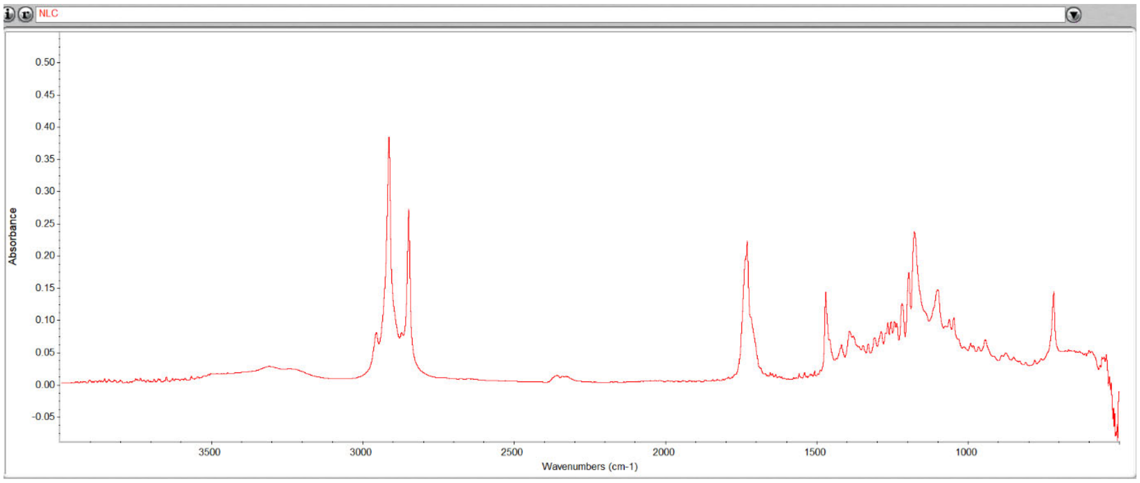
NLC
0.50
0.45
0.40
0.35
0.30
0.25
0.20
0.15
0.10
0.05
0.00
-0.05
Absorbance
3500
3000
2500
2000
1500
1000
Wavenumbers (cm-1)

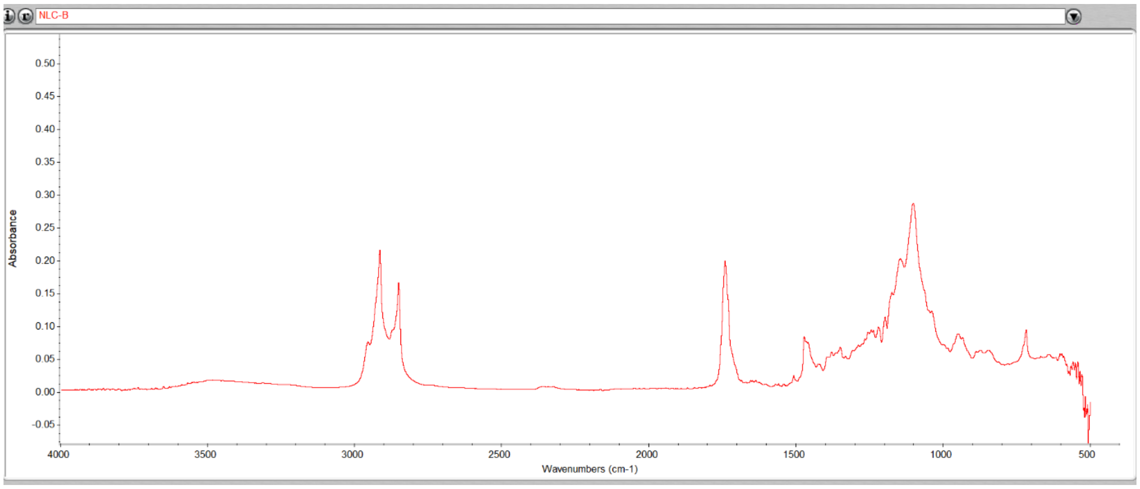

TEM micrographs

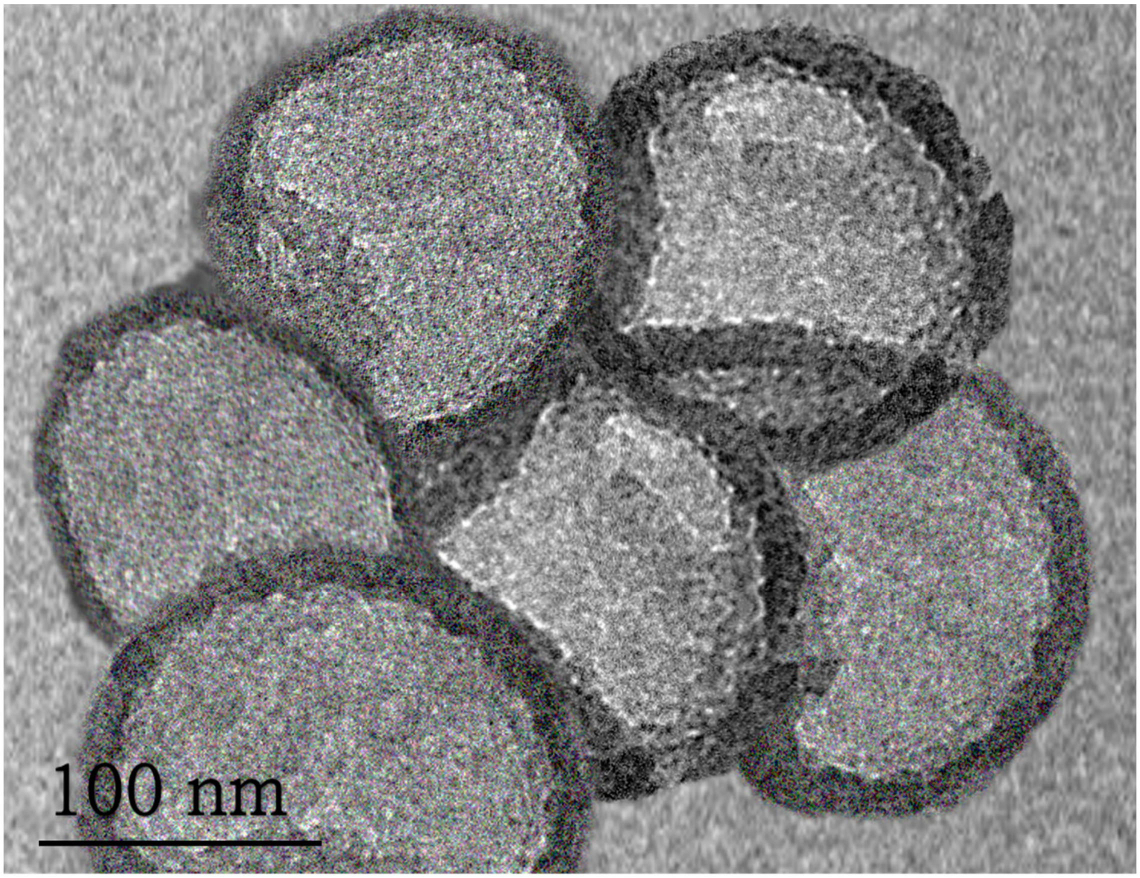
100 nm

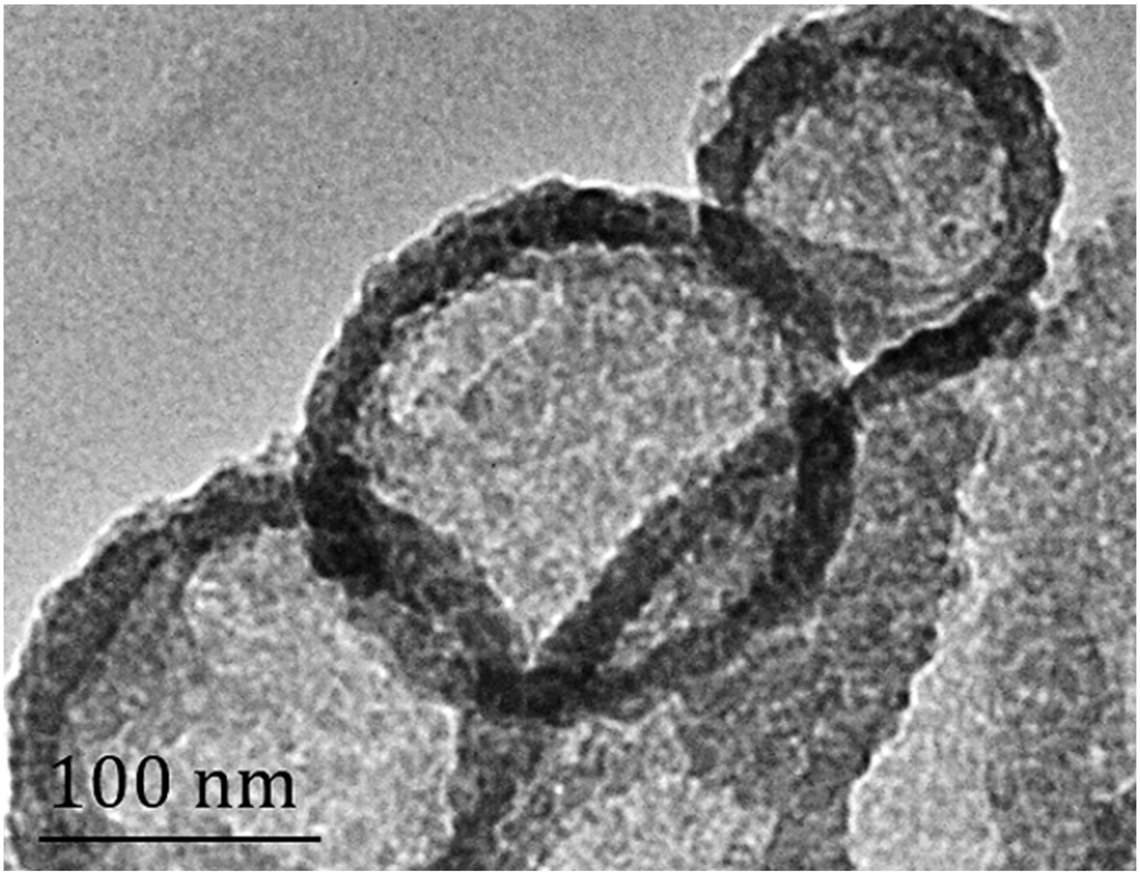
100 nm
